# Comparison of DNA Quantification Methods for Next Generation Sequencing

**DOI:** 10.1038/srep24067

**Published:** 2016-04-06

**Authors:** Jérôme D. Robin, Andrew T. Ludlow, Ryan LaRanger, Woodring E. Wright, Jerry W. Shay

**Affiliations:** 1 Department of Cell Biology, 5323 Harry Hines Boulevard, UT Southwestern Medical Center, Dallas, TX 75390-9030, USA; 2 Center for Excellence in Genomics Medicine Research, King Abdulaziz University, Jeddah, Saudi Arabia

## Abstract

Next Generation Sequencing (NGS) is a powerful tool that depends on loading a precise amount of DNA onto a flowcell. NGS strategies have expanded our ability to investigate genomic phenomena by referencing mutations in cancer and diseases through large-scale genotyping, developing methods to map rare chromatin interactions (4C; 5C and Hi-C) and identifying chromatin features associated with regulatory elements (ChIP-seq, Bis-Seq, ChiA-PET). While many methods are available for DNA library quantification, there is no unambiguous gold standard. Most techniques use PCR to amplify DNA libraries to obtain sufficient quantities for optical density measurement. However, increased PCR cycles can distort the library’s heterogeneity and prevent the detection of rare variants. In this analysis, we compared new digital PCR technologies (droplet digital PCR; ddPCR, ddPCR-Tail) with standard methods for the titration of NGS libraries. DdPCR-Tail is comparable to qPCR and fluorometry (QuBit) and allows sensitive quantification by analysis of barcode repartition after sequencing of multiplexed samples. This study provides a direct comparison between quantification methods throughout a complete sequencing experiment and provides the impetus to use ddPCR-based quantification for improvement of NGS quality.

Next Generation Sequencing (NGS, also called High Throughput Sequencing) has facilitated a surge of new discoveries, from genotyping cohorts of patients for the identification of disease-associated mutations to the exploration of cellular processes and chromatin landscapes^1^,^2^,^3^. Whole-genome sequencing^4^,^5^,^6^,^7^,^8^ has become the method of choice for acquiring large data sets for broad genomic analyses, including the International Network of Cancer Genome Projects^9^, Cancer Genome Atlas Research Network^10^, the Integrated Genomic Analyses of Ovarian Carcinoma^11^, and the Encyclopedia of DNA elements project (ENCODE)^12^. With such large data sets and discoveries hinging on this technique, it is imperative that each step of NGS is optimized, accurate, and cost-effective.

Current methods for quantifying DNA library preparations for NGS use a variety of techniques (Table 1): UV absorption (e.g., Nanodrop); intercalating dyes (e.g., QuBit; Invitrogen, SYBR Green); 5′ hydrolysis probes (e.g., Taqman^®^) coupled with real-time quantitative PCR (qPCR; Kapa Biosystem) or droplet digital emulsion PCRs (ddPCR; Bio-Rad). For most of these methods, large amounts of DNA are required to provide accurate titration. The primary reason why sequencing platforms require micrograms of library preparations (Illumina recommendation) is to ensure that enough material is available for quantification^13^,^14^,^15^. The amount of DNA needed for sequencing (12 pM per lane on average) is about 1000-fold less than what is needed for quantification. For certain libraries, this amount (i.e., microgram) of DNA is not available without excessive PCR amplification.

The generation of micrograms of DNA by PCR is a sensitive process subject to many variations. Increasing the number of PCR cycles leads to loss of sequence heterogeneity, potentially resulting in variable sequencing quality^2^,^13^,^15^; distortion of the original ratios/proportions of sequences and finally causing the loss of rare input molecules^16^. Also, sequence heterogeneity might be modified by PCR amplification altered by over amplification of shorter fragments, GC:AT content, and DNA/Taq polymerase bias^17^. Hence, most would agree that unnecessary amplification for the sole purpose of NGS library quantification should be avoided and cycle number should be minimized to prevent biases^18^.

Our ability to reduce the number of cycles of PCR is hampered by the fact that current NGS library quantification methods are inadequate. To improve NGS library quantification, several methods have been developed. White *et al.* developed an original technique which utilizes 5′ hydrolysis probes and digital emulsion PCR using a chip to create ~1000 individual PCR reactions per chip^19^. In brief, they incorporated the complementary sequence of a 5′ hydrolysis probe to the forward primer sequence such that a hydrolysis probe can be used to detect library PCR products following end-point PCR (Fig. 1). This strategy and recent advances in emulsion PCR instruments allow one to combine accuracy, sensitivity, and scalability in library quantification. Droplet digital PCR devices, such as the QX100^®^ ddPCR System (Bio-Rad Laboratories) can generate ~20,000 droplets and help ensure accurate quantification of DNA molecules from library preparations. Droplet digital PCR (ddPCR) generates thousands of droplets containing template DNA, partitioned and amplified within each individual droplet, allowing for smaller sample reaction sizes and also preserving rare molecules. After PCR, droplets are counted with a droplet reader and scored as either fluorescent or not, a binary readout is produced for the 20,000 droplets (hence the term digital). The ratio of PCR positive to negative droplets is calculated, and Poisson statistics corrects for the possibility of having one or more template molecules per droplet. The resulting quantification of input DNA molecules is given as a concentration [molecules of target per microliter of input PCR], and this concentration is easily converted to total number of input molecules of a given sample. DdPCR simplifies and improves the process of determining the number of input DNA molecules in a given sample compared to methods using standard curves and qPCR.

In this work, we combined the power of 5′ hydrolysis with ddPCR to achieve quantification of NGS libraries, and compared this method with samples quantified by other traditional methods. We describe the adaptation of the universal template probe digital PCR method to the BioRad QX100^®^ ddPCR platform for the quantification of sensitive low abundance NGS libraries. We extend our report with the adaptation of the UPL technology (Universal Probe Library; Roche) to the same platform and provide a comparison between the current NGS titration methods. The methodology described here provides sensitivity and stability not achieved by other techniques. By directly comparing identical samples through a full sequencing run as a proof of principal pilot series of experiments, we present an end-point analysis that was missing regarding NGS quantification strategies.

## Results

### TaqMan^®^, UPLs^®^, Tail- based strategy give comparable results in ddPCR

We first determined if Universal Probe Technologies (UPL^®^, Roche) worked comparably to traditional 5′ hydrolysis probes (Taqman^®^) in a ddPCR set-up. Using two genes, we compared, with dilution of cDNA inputs, the level of detection in ddPCR (Figure SI, SII). The first comparison was made between TaqMan^®^ and UPL^®^ probes (Figure SI) with primers detecting exons 7 and 8 of *hTERT* (human telomerase reverse transcriptase, NCBI Gene ID: 7015) from cDNA. In our experiments, universal probes were as effective as traditional 5′-hydrolysis probes as shown by the strong associated correlation (goodness of fit) found between the linear regressions (R^2^ = 0.9999; p < 0.0001).

**Figure 1:**
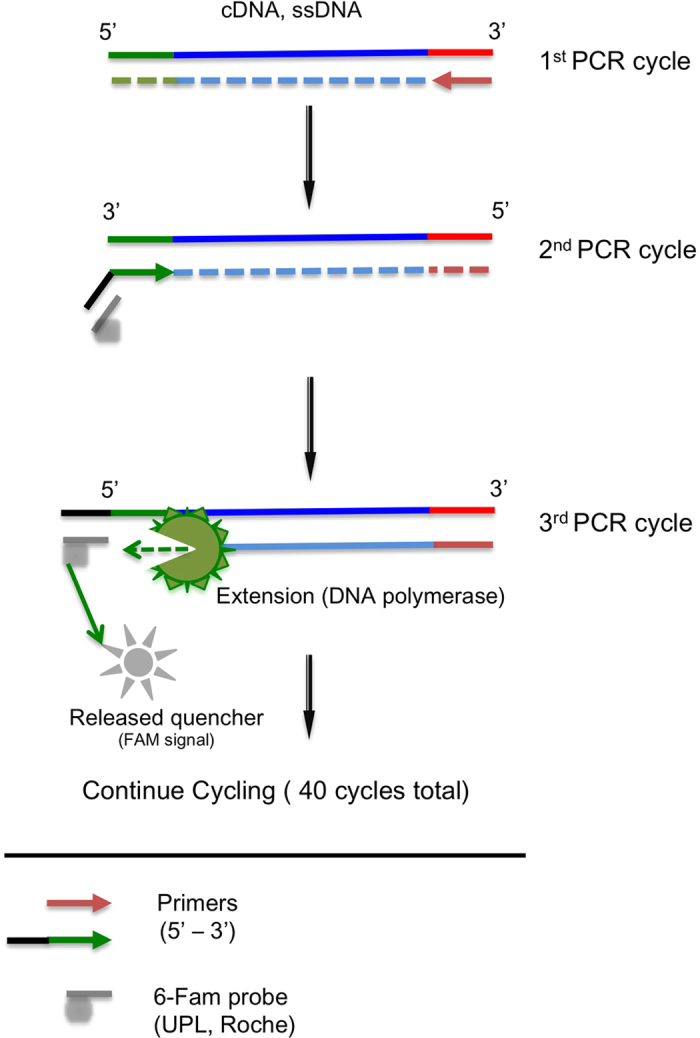
Overview of the ddPCR-Tail strategy using UPLs^®^ (a 5′probe-tailed primer). At the 5′ end of the forward primer, a short sequence complementary to a UPL^®^ (Roche) was added. The sequence generated during the first PCR cycle (faint colored strand) will allow the tail to be incorporated in the second cycle (black strand). Thus, during the next PCR cycle (3^rd^ round), the 5′ to 3′ exonuclease activity of the DNA polymerase will cleave the probe (in grey) creating fluorescence signal (positive droplets). This tail-probe strategy is highly adaptable since knowledge of the sequences between the primers is not necessary.

To further confirm that Roche probes (Universal Probes Library®; LNA modified 8–9 DNA-oligomers) work in droplet digital PCR, we performed an additional test on a common reference gene, *HPRT* (hypoxanthine-guanine phosphoribosyltransferase, NCBI Gene ID: 3251). As a proof of principle, we tested a 5′ tail or ‘universal probe’ strategy (referred to as droplet digital PCR-Tail or ddPCR-Tail)^19^. In this strategy, the complimentary sequence to a universal probe sequence (Roche Universal Probes®) is added to the 5′ end of the forward primer (outlined in Fig. 1 and SII). In this situation, one can accurately quantify the input material (i.e., library quantification for NGS or mRNA level) without knowledge of the sequence between the forward and reverse primers. To validate this concept, we first tried the ‘ddPCR-Tail’ strategy in a simple quantification system (i.e., gene expression: one amplification product). We utilized reference gene primers for *HPRT* with an internal Universal Probe Library #22 (UPL#22) to the primers and a 5′ tail probe of unique sequence (UPL#52). We used serial dilutions of cDNA made from total RNA to determine if the tail and internal probe strategy gave similar concentration values. We observed a strong correlation between the two techniques, as revealed by the two linear regression curves (goodness of fit; R^2^ = 0.9923, p < 0.0001), indicating similar detection between the different strategies (TaqMan^®^, internal and tailed UPLs^®^) (Figure SII).

### Titration of NGS libraries with ddPCR-Tail gives absolute input molecule counts

**Figure 2:**
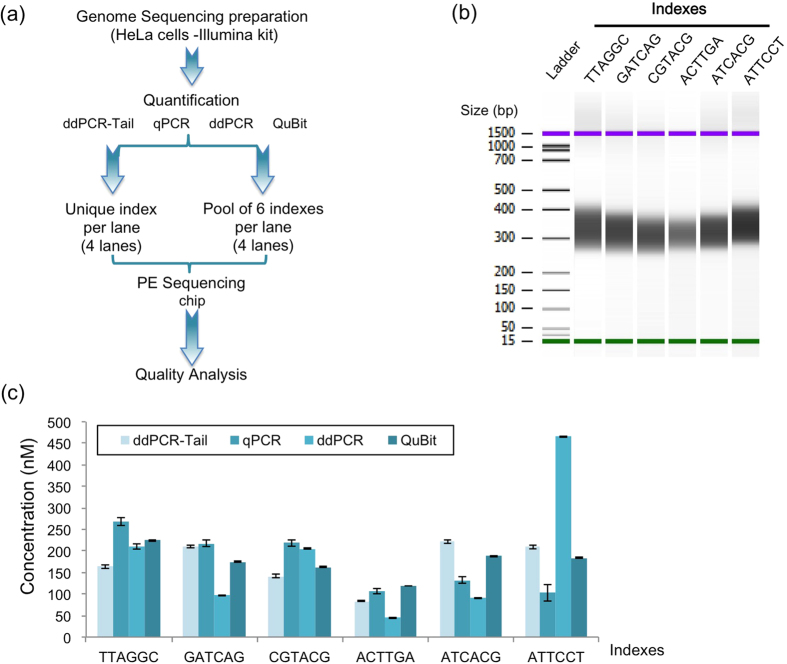
Comparison of four titration techniques for NGS. (**a**) Diagram of the Next generation sequencing (NGS) experimental design. Using four quantification methods, we titrated DNA libraries prepared from HeLa cells, following manufacturers’ instructions. Six different indexes were added at the amplification step. Of the eight lanes on the NGS flowcell, four lanes were used to compare each method (unique index lane) and four lanes for pooling accuracy (pool of six indexes per lane). (**b**) Bioanalyzer image of the libraries, showing a homogenous smear of DNA from 280 to 450 bp. All six indexes show similarly average sizes. (**c**) Result of the quantification for all indexes using QuBit, qPCR, ddPCR and ddPCR-Tail approaches, respectively. All quantifications (except ddPCR-Tail) were done following manufacturer instructions (Invitrogen, BioRad, and KapaBiosystem). The ddPCR-Tail strategy used the same apparatus as the ddPCR with slight modifications (50 nM primers; three-step PCR-annealing at 65 °C for 30 seconds, extension time of 30 seconds at 72 °C). Experiments done in triplicate, mean calculated using dilution curve (average of 12 values per quantification, 6 for QuBit). Mean ± SD shown.

We applied the ddPCR-Tail strategy to NGS quantification by adding a 5′ sequence to the PE universal primer 1.0 (Illumina) and compared the ddPCR-Tail method to the other techniques used for NGS DNA library quantification. The same sample was quantified (with 6 different indexes) with commercial kits using QuBit, qPCR, ddPCR, and ddPCR-Tail (Fig. 2). Molarities were calculated according to the respective technology and corrected with a standard curve when required (e.g., KapaBiosystem for qPCR). All quantification methods successfully estimated libraries in the same concentration range (50–250 nM). For one index (ATTCCT), the molarity was different with the ddPCR Kit (465 nM), an outlier that potentially reveals an incompatibility between the probes used in that kit and that particular index sequence. Nevertheless, taken separately, all indexed-libraries measurements are significantly different when comparing each method (Fig. 2c). Indeed, adjusted p values calculated using the Sidak’s multiple comparison test (alpha = 0.05) revealed p values below 0.0001 with one exception (GATCAG: ddPCR-Tail vs. qPCR; adjusted p value = 0.0956). Therefore, only the outcome of the sequencing result will clearly determine which quantification method was the most reliable. The use of ddPCR or ddPCR-Tail does not require the back-calculation of the library against an average size determined by a Bioanalyzer assay (Fig. 2b), making ddPCR-based quantifications less time- and reagent- consuming. Quantification with QuBit or qPCR requires the additional equipment in order to determine the molarity of libraries with further calculations. qPCR and QuBit give relative measures while ddPCR ones are absolute.

### ddPCR-Tail gives sensitive and reliable titration for NGS

**Figure 3:**
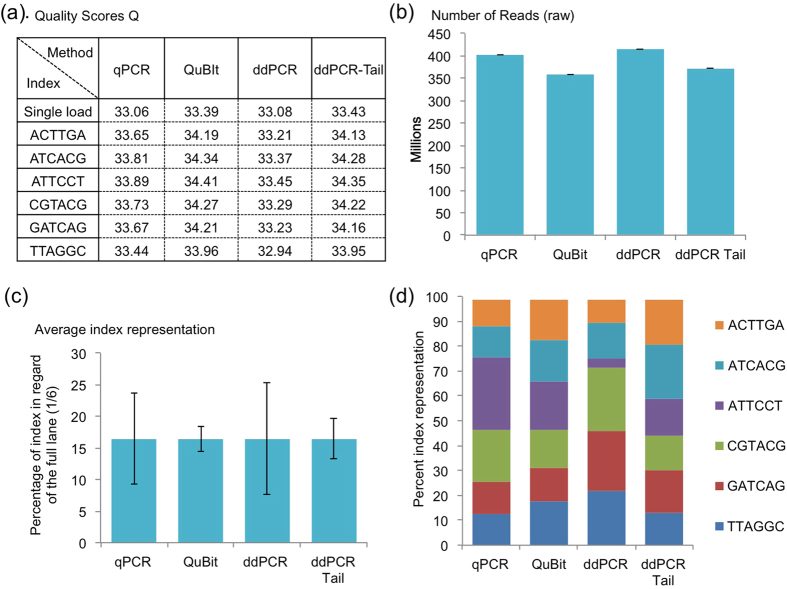
Quality analysis of the 6-index pool. (**a**) Table of the average quality (Q) scores from the four separated lanes (reported as single load) and the pooled six indexes across the four titration methods (all from read 1; total of 7 average Q score for each titration). Quality scores are fairly good (with 34 being the gold standard for QE results), with better than optimum results from the QuBit and ddPCR-Tail strategies (mean ± SD, QuBit = 34.23 ± 0.15; ddPCR-Tail = 34.18 ± 0.13). (**b**) The total number of reads (PF reads) available from the sequencing results (from un-pooled and pooled experiment), i.e represents the number of reads passing basic control and identified as unique clusters. For each assay, un-indexed reads represents less than 2% (qPCR = 1.55%, QuBIT = 1.54%, ddPCR = 1.32%, ddPCR-Tail = 1.49% respectively). All methods gave greater than 350 million reads: qPCR and ddPCR (402, 414); QuBit and ddPCR-Tail (357, 372). Mean ± SD shown calculated from duplicate experiments. (**c**) The average index representation Mean ± SD shown. (**d**) The specific repartition of the six indexes within the four different lanes, from the four different titration methods.

Using four lanes of a Paired-End sequencing (PE-Seq) experiment, we pooled the 6 indexes based on the titration method used and sequenced them (PE-100nt, HiSeq2500; Illumina). Our purpose was to compare different titration methods, thus the focus was directed towards the quality and repartition of the reads rather than the actual sequences contained in the reads (Fig. 3, SIII). We first examined the FastQ data using Q scores. The Q score is a value indicating the probability of a base (nucleotide) being called incorrectly; Q scores are given to each nucleotide of the read (calculation is done by a phred-like algorithm, similar to that originally developed for Sanger sequencing experiments)^20^. Globally, a Q score of 20 represents an error rate of 1 in 100, with a corresponding call accuracy of 99%. In PE-seq, a Q score is “good” when above 34 (error rate of about 1 in 2500; 99.96% accuracy). In these experiments, we observed that QuBit and ddPCR-Tail gave slightly better Q scores (one-way ANOVA; p < 0.0001 with no significant differences only between QuBit and ddPCR-Tail using the correction for multiple comparisons) with an average of 34 and respective standard deviation (SD) of ± 0.15 and ± 0.13. In comparison, ddPCR and qPCR average a Q score of 33 (accuracy of 99.95%), with SD values of ± 0.17 and ± 0.15, respectively (Fig. 3a). However, the Q score value is incomplete without considering the total number of reads (Fig. 3b). DdPCR and qPCR methods produced more reads (means ± SD; ddPCR = 414 ± 2.42 and qPCR = 402 ± 27 million PF reads; i.e., reads passing illumina’s filter), suggesting potential overloading of the lane leading to difficulty to separate clusters, hence a lower Q score. This could be explained by an underestimation of the libraries concentration, a consequence of PCR amplification failures, or probe/enzyme related issues favoring specific sequences in both titration assays.

Next, using our indexed approach, we evaluated the reproducibility of titration methods regardless of the indexing sequence, since all libraries are identical (from the exact same preparation) and represent technical replicates. Ideally, a reliable and sensitive technique will allow the indexing of multiple samples in a homogenous fashion (equal ratios). Optimal balancing of the indexed libraries would result in a ~16% per indexed library (approximately one sixth of 100%). QuBit and ddPCR-Tail provided the most stable results (Fig. 3c), with the least amount of variability between measures relative to a lower variation between DNA indexed libraries (reported by lower standard deviation respectively ddPCR-Tail SD ± 3.1; QuBit SD ± 1.9; qPCR SD ± 7.2 and ddPCR SD ± 8.9). However, when representing the data to appreciate the true repartition between index sequences (Fig. 3d), we observed that some indexes are vastly over-represented. For example, the ATTCCT index is over-represented with qPCR titration, whereas it is almost completely absent with the ddPCR titration. These disparities could be linked to either an issue with the index sequence itself or a partial failure of the titration. The ddPCR-Tail and QuBit assays achieved the best results with regard to index equilibrium allowing one to multiplex sequencing experiments in an accurate and balanced fashion. Therefore, this can reduce bias and potentially minimize the cost of sequencing per sample by loading more indexed libraries into the same lane with confidence that each library will be equally sequenced.

**Figure 4:**
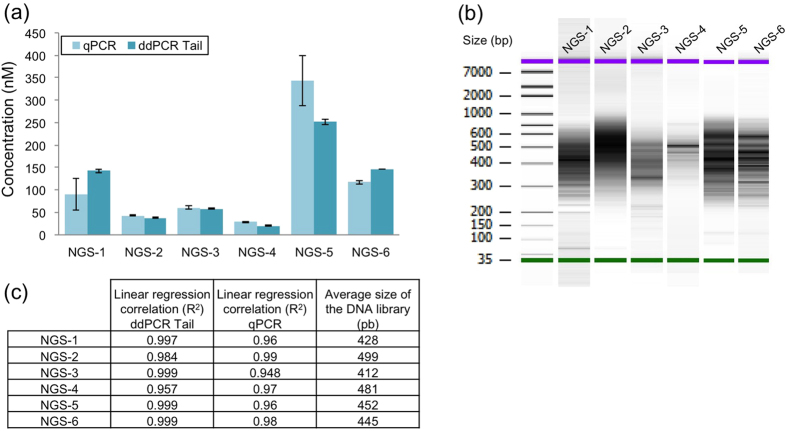
Titration of six NGS libraries with low abundance with qPCR and ddPCR Tail. (**a**) Comparison of two methods for low abundance NGS libraries, titration results using qPCR and the ddPCR-Tail system. Assay done in triplicate and mean calculated using dilution correction factors (average of 18 values per sample). Mean ± SD shown. (**b**) Bioanalyzer image results used to calculate final molarity for the qPCR measurement, algorithm provided by KapaBiosystem using the average size of the NGS library combined with the qPCR standard curve. (**c**) All libraries were successfully quantified with high confidence (linear regression all in 0.9), ddPCR Tail strategy showed a better overall linearity regardless of the heterogeneity of the libraries.

To assess the sensitivity of the ddPCR-Tail method, we quantified some challenging NGS libraries with low amounts of DNA, wide heterogeneity in DNA sizes and traces of primer dimers. We compared the results to the most commonly used quantification method, qPCR. We analyzed complex libraries prepared for Hi-C sequencing (Fig. 4a,b), an NGS-based technique to investigate genome-wide chromatin structure^21^,^22^. Due to the numerous enzymatic steps required for the assay, the amount of prepared DNA is limiting. One must have a very sensitive means for quantifying the library prior to NGS to reduce PCR bias^18^. In this case, a poor library will result not only in fewer reads, but also in a lack of representative sequences. Rare ligations might be overlooked and relative levels of interactions might be distorted due to the bias introduced during PCR amplification^18^. The ddPCR-Tail method provided equivalent quantification results as the one calculated from the qPCR method in 4 out of 6 libraries (Fig. 4a; NGS-2, p = 0.99; NGS-3, p = 0.99; NGS-4, p = 0.94; NGS-6, p = 0.99 respectively, adjusted p values calculated using Sidak’s multiple comparisons test; alpha = 0.05). This compliments our earlier observation that ddPCR-Tail is equally as reliable as qPCR for DNA quantification.

Finally, we addressed the stability of the technique in heterogeneous Hi-C libraries (Fig. 4c). DdPCR-Tail gives better linear regression correlations (Student T-test p = 0.04; Fig. 4c) and smaller standard deviations compared to qPCR (Fig. 4a; NGS-1 and NGS-5). Two potential sources of error that could explain the large standard deviations observed in NGS-1 and NGS-5 include intra-experiment variations (i.e., enzyme activity) and technical variability (pipetting).

However in order to minimize the technical-induced variability, and increase the strength of the statistical analysis, we performed the assay in triplicate of triplicates (triplicate of each dilution points, in 3 different assays for a total of 18 values per sample minimum), the two libraries quantification are statistically different and more variable when compared to the result provided by the ddPCR-Tail (Fig. 4a; NGS-1 adjusted p value < 0.0001; NGS-5 adjusted p value p < 0.0001, Sidak’s multiple comparisons test). DdPCR-Tail provides quantifications that are more reproducible compared to qPCR as shown by the six different HiC libraries titrations. Since ddPCR-Tail is extremely sensitive, as evidenced by the large dilution used in order to quantify libraries, one could apply this method to titrate libraries with minimal PCR amplification: moving the limitation of DNA material needed for NGS experiments towards the capacity of the sequencer.

## Discussion

We provide here an evaluation of a highly accurate platform for quantification of high throughput sequencing libraries. We also provide evidence for the utility of the universal primer strategy for detection and quantitation of gene expression. Further, we show that UPLs^®^ is as efficient as traditional 5′ hydrolysis probes on the Bio-Rad droplet digital QX100^®^ system. Importantly, we have compared different NGS library quantification strategies and shown that ddPCR is as sensitive and accurate as other commonly used DNA quantification strategies prior to NGS and uses less input DNA.

A reliable and accurate DNA quantification strategy will permit investigators to fully utilize sequencer capacity, thus reducing costs of sequencing even further. Presently, a second-generation sequencer that is loaded with too much DNA or with unbalanced indexed NGS libraries may result in a complete loss of the sample (i.e., loss of one specific index) because of errors associated with titration techniques. The ability to index multiple samples (theoretically infinite but typically limited to 24 per lane) is then completely dependent on the reliability of the titration method, leading in most cases to an under-use of the capacity of the NGS flowcell and an unnecessary number of reads/high coverage for genotyping. This caveat consequently increases the cost of sequencing per sample and time required to complete analysis of large-scale experiments, such as large cohort-whole genome sequencing.

In this study, using a whole genome-sequencing library, QuBit performed comparably to our ddPCR-Tail and better than the current PCR and ddPCR kits. When comparing the cost and time required for the different techniques, QuBit is fast and requires an apparatus that is far cheaper than a real-time quantitative PCR or a droplet digital PCR machine. However, our libraries were concentrated, homogenous, and had no detectable trace of primer dimers (Fig. 2b), an unrealistic situation for most library preparations. Most NGS libraries are prepared from valuable limited samples of various qualities. When input material is limiting, preparation can lead to libraries as faint and heterogeneous as the ones shown in Fig. 4. With no absolute quantification, qPCR and QuBit methods highly depend on the average size determined by the Bioanalyzer. The bioanalyzer estimates an average size of library, based on DNA intensities detected at specific sizes. Thus, in complex libraries, primers and contaminants will distort the true average size and will generate inaccurate values. For qPCR and QuBit methods, uncertainty in the average size of the library will affect the yield of DNA loaded onto the flowcell. Since we used a massive amount of input DNA (5 μg per index; Fig. 3) we did not observe these biases. For these reasons (i.e., no primer dimers, homogenous size, high quality DNA), the QuBit quantification was un-biased of any non-amplifiable sequences (primer dimers) and was at least equivalent to the performance of our ddPCR-Tail assay (Fig. 2).

In this proof of concept experiment, we compared the titration methods using an indexed strategy on a NGS flowcell (Fig. 4). Ideally, in order to gain strict statistical power over quality of sequencing and fully extend our comparison between sequencing results (depth of reads, cluster generation, etc), the same assay should be repeated on multiple independent HiSeq runs. However, our goal in these analyses was to provide a comparison between titration techniques and to provide evidence for robust quantification of NGS libraries using ddPCR. Hence, we performed a sequencing run with a single flowcell loaded with replicate libraries (Fig. 2a). First, with four lanes loaded with the same library quantified with each method. Second, we used the remaining four lanes for multiplexed libraries. This design generated sufficient preliminary data supporting that our quantification method with ddPCR was valid and more accurate compared to others commonly used methods for NGS library quantification strategies (Fig. 3).

We first considered the different indexes as simple replicate experiments. This decision is justified by the fact that only the index sequences vary from those samples produced under the exact same conditions. Only the primer added when producing the libraries were different (making those samples technical replicates). This approach provides insight about the reliability of the titration methods and suggests that QuBit and ddPCR-Tail are better compared to other methods tested (Fig. 3c). Both techniques reduce the variations of library quality within a lane (due to titration) that could affect the overall Q-scores of the full lane. Commonly, in order to avoid loss of quality and data, one chooses to pool a limited number of libraries in order to compensate for potential error in titration that would result in a complete loss of the sample (e.g., over-representation of one sample over others resulting in unusable data).

Excessive amplification in sensitive NGS assays (HiC, 4C, ChIP-Seq, ChIA-PET) is only required due to the poor precision of classic titration methods at very low concentrations. Thus, better sensitivity is crucial for decreasing the number of duplicate reads (that are likely false values) found when using extensive PCR for preparing the experimental samples. Loading between 8 to 12 pM per lane is optimal; any additional PCR cycles beyond this minimal amount of DNA leads to the introduction of bias (i.e., Taq polymerase favors certain sequences based on GC:AT content, secondary structure of the DNA and size^18^) and the loss of sensitivity. In this work, because dilutions used to quantify the NGS libraries with ddPCR-Tail are high (i.e., from 1:1,024,000 to 1:262,144,000) and resulted in successful sequencing experiments (Fig. 3), one can consider future experiments using libraries with reduced PCR cycles. This is an encouraging observation to apply in methods where the limiting factor is the amount of DNA to be loaded on the flowcell (8 to 12 pM per lane).

With the exception of emulsion digital PCR, all methods are based on mass derived standards, which in turn necessitates the calculation of molecule numbers based on fragment length distribution, resulting in additional bias depending upon the methods used to determine the standard’s mass^18^,^19^. Interestingly, if required, average size determination of sample is also achievable by ddPCR using the intensity levels of the droplets in a different PCR setting (i.e., concentration range of template and PCR profile)^23^. Q-PCR techniques require triplicates of each point and dilution, as well as triplicates of the given standard. Due to inherent variation between plates, a standard curve and PCR efficiency calculation is required in each run. Combining the PCR efficiency (linear regression from the standard curve), the known standard concentration and size leads to a formula that is used to convert the Ct values obtained from the diluted library to DNA quantity. In contrast, ddPCR output is the number of molecules detected in the sample. Converting ddPCR results into a molarity is easily done and does not require the use of standards, regression curves or the average size of the library given by a second method (bioanalyzer). This in turn saves time and space on quantification plates and may facilitate high-throughput titration of libraries.

## Conclusions

Using droplet digital PCR, our results provide strong evidence that standard curve-free nucleic acid quantification is achievable for NGS libraries and gene expression. DdPCR-Tail is highly sensitive and proved to be comparable, if not better, than current methods (qPCR), a finding that is in agreement with known theoretical assessments (measurement of molecule numbers). Nevertheless, our work also reports comparable quality results from the QuBit assay, suggesting that this method should be considered over other methods that are costly and time-consuming, such as when one has clean and homogenous library with no primer dimer problems.

In summary, this work offers a comparative analysis of NGS titration methods, and reveals that ddPCR-based assays along with QuBit allows one to use the capacity of second generation sequencing to its fullest.

## Materials and Methods

### Study design

Using a whole genome NGS assay, we compared quantification tools commonly used in library titration: fluorometry (QuBit), qPCR, ddPCR and ddPCR-Tail (workflow in Fig. 2A). In brief, a full Paired-End sequencing (PE-Seq) flowcell was used (8 sequencing lanes). Four lanes were loaded with a single library preparation to compare each quantification method. The four remaining lanes were used for multiplexing experiments. Different tag sequences (six indexes) were added during the last step of the library generation process, allowing the samples to be pooled together in one lane (multiplexed) and then using bioinformatics tools (CASAVA 1.8.2, Illumina) separated after sequencing. Each tagged library was quantified separately using the four methods. Then libraries were combined into pools of multiplexed libraries (from each method) and finally re-quantified. In short, for the qPCR, the traditional set up was performed (triplicates, for intra variation) for 5 dilution points and run three times (three different assays, for inter variation). For all libraries a minimum of 18 (up to 27) values were used to get an estimation of the quantification. For the ddPCR, according to the manufacturer’s instruction (BioRad) we used a 3 point dilution, in duplicate (5 point dilution and no duplicate for the ddPCR-Tail). The ddPCR-based assays were then run in triplicate for a total of 18 values per libraries (15 values for ddPCR-Tail).

Finally, the pooled libraries were run on separate lanes, with one lane per quantification method (six indexes per lane). This provided an opportunity to test the reproducibility of the different methods and the robustness of the assays between different index sequences (Fig. 2A).

### RNA extraction and gene expression analysis

RNA was extracted from 1 × 10^6^ HeLa cells (obtained from the ATCC, Manassas, VA) using a column-based kit (RNeasy Plus, Qiagen) following the manufacturer’s instructions. RNA was quantified using a Nanodrop and 1 μg of total RNA was reverse transcribed with a first-strand cDNA synthesis kit (iScript, BioRad) using an optimized combination of random hexamers and oligo-dT. Following cDNA synthesis, the samples were diluted one to four in water and stored at −20 °C until further use. For PCR primer linearity tests, a 7-point, 1:4 serial dilution of the samples was prepared (i,e: 1:4; 1:16; 1:64; 1:256; 1:1,024; 1:4,096; 1:16,384) and run with a no-template control (NTC). For gene expression analysis, mRNA quantification was pursued using manufacturer’s instructions (ddPCR, BioRad). In brief, 1 μl of diluted cDNA was used in a 19 μl reaction composed as follows: 10 μl of ddPCR Master mix (2x), 250 nM of each primers and either 0.1 μL of the chosen UPL (10 μM) or 0.1 μL (10 μM) of the Taqman probe, the mix was brought up to a final volume of 19 μl with DNase free water. After droplet generation, the sealed plate was transferred in a thermo-cycler with the following cycles: 95 °C heat activation for 10 min followed by 40 cycles of 30 seconds at 95 °C for denaturation and 1 min at 60 °C for annealing-extension steps. At last, the PCR reaction was stopped with a final deactivation at 98 °C for 10 min. The plate was then loaded into the ddPCR QX100 reader.

### NGS library preparation

For the genome-sequencing assay, DNA was extracted from HeLa cells using a high salt extraction technique 24. Briefly, cells were lysed overnight at 37 °C (lysis buffer, 50 mM Tris-HCl pH7.4; 20 mM EDTA; 1% SDS and Proteinase K at 200 ng/ml). After proteinase K inactivation (65 °C for 10 min) samples were RNaseA treated (100 ng/ml; 37 °C for 2 hours). DNA extraction was performed by adding 6 M NaCl to the solution, which was then vigorously shaken for 30 seconds prior to a 7 min centrifugation at 10,000xg. Next, the supernatant was transferred to a new tube and completed with a two-fold volume-to-volume solution of 100% ethanol. A second spin (4 °C for 30 min, 10,000xg); was then performed to precipitate the DNA, the pellet was washed once with 70% ethanol, spun down, air dried and re-suspended in TE buffer.

After quantification (Nanodrop), 5 μg of DNA for each indexed library (n = 6) was used to prepare the NGS whole-genome sequencing assay following the manufacturer’s instructions (TruSeq^TM^ Sample Prep Kit-v2, Illumina). Shearing DNA was performed using adaptive focused acoustic (AFA) technology. We used a total processing time of 80 seconds per sample in AFA microTUBE (AFA™Focused-ultrasonicator conditions for the S-Series; Covaris). Prepared libraries (after PCRs amplification) were analyzed on a Bioanalyzer (Agilent, Fig. 2B) and 12 pM were loaded per lane into the sequencer. A total of six indexed libraries were prepared from the same DNA extract.

For high-resolution chromosome conformation capture (Hi-C) libraries (Fig. 4), DNA was prepared using a previously published protocol^25^,^26^. A total of six different libraries were produced from six different cell lines (all from myoblasts isolated from different biopsies)^22^.

### NGS library titration

DNA was quantified by four separate methods. All samples were analyzed with a Bioanalyzer (Agilent) DNA high-sensitive chip, which gives information on the size distribution (Figs 2B and 4). This step is required for the accurate back calculation of molarity from qPCR and QuBit quantifications.

### QuBit

Samples were diluted to a 5 ng/μl concentration (estimated by the bioanalyzer results) and quantified using the QuBit dsDNA HS Assay kit and the QuBit 2.0 fluorometer following the manufacturer’s instructions (Life Technologies). Prior to measurement, a five-point calibration curve was established using the supplied standard. One-microliter of the 5 ng/μl solutions were diluted 200-fold in Qubit assay dilution buffer and measured on the fluorometer. Samples that fell below the limit of detection were re-quantified using lower dilutions. Concentrations provided by QuBit were used to calculate the molarity of the initial sample, correcting for the dilution factor and converting to molarity using the average size of the library as detected by the bioanalyzer (Fig. 2). Measures were done in duplicates.

### qPCR

Quantification of libraries using qPCR was done using a commercial kit (KapaLibrary quant kit, KapaBiosystem) and a LightCycler 480 (Roche). Following the manufacturer’s guidelines, dilution series were made for all libraries and run in parallel with the provided standards (a series of pre-made dilutions of a 452nt product). A set of five dilutions (1:1,000; 1:2,000; 1:4,000; 1:8,000, 1:16,000) was made for all libraries and compared to six standards (respective concentrations of 20, 2, 2 × 10^−1^, 2 × 10^−2^, 2 × 10^−3^, 2 × 10^−4^pM). All samples and standards were run in triplicates and subjected to PCR cycles as follows: 95 °C heat activation for 5 min followed by 35 cycles of 95 °C denaturation for 30 seconds and annealing-extension at 60 °C for 45 seconds. The assay was done in triplicate, giving a maximum of 45 values per library. However, after exclusion of obvious outliers (e.g., high Cts, replicate data point > 0.2) this number felt around 20 values per library. The standard curve was used to calculate the PCR efficiency and subsequently the molarity of the libraries after correction, using the manufacturer’s recommended curve to scale the quantification based on average fragment size determined on the Bioanalyzer.

### ddPCR

Similarly, for ddPCR titration, we used a series of dilutions made from the initial samples (1 × 10^−6^; 1 × 10^−7^; 1 × 10^−8^). Then, according to the kit instructions (ddPCR™ Library Quantification Kit for Illumina TruSeq, BioRad), we prepared mixes of 20 μl reactions per sample as follows: 10 μl of 2x ddPCR Supermix; 1 μl of 20x ddPCR library reaction assay; 5 μl DNAse-free water completed with 4 μl of the diluted sample to reduce pipetting errors. Droplets were generated, transferred to a thermocycler and submitted to the following cycling program: 95 °C heat activation for 10 min followed by 40 cycles of 30 seconds at 95 °C for denaturation and 1 min at 60 °C for annealing-extension steps. Finally, the PCR reaction was stopped with a last step at 98 °C for 10 min. The plate was then transferred and loaded into the ddPCR QX100 reader. Results obtained correspond to the number of molecules in one reaction. After dilution corrections, the number of molecules was converted into molarity. All samples were run in duplicate in three separate assays for a total of 18 values per libraries.

### ddPCR-Tail

We combined UPLs technology (Roche) with ddPCR technology (BioRad), by using a set of primers (Table S1) where the forward primer had an additional nine nucleotides on the 5′ end corresponding to a reverse and complemented UPL probe sequence (UPL#52: 5′CTCCTCCC3′). UPLs^®^ are short oligonucleotides (8–9 mers) made of mixed LNA and DNA 5′ hydrolysis probes (Roche). Additional modifications were made from the gene expression ddPCR probe based protocol. To summarize, one μl of the diluted DNA library was used in a reaction as follows: 10 μl of ddPCR Master mix (2x), 50 nM of each primers complemented with 0.1 μl of UPL#52 in a final volume of 19 μl with DNAse free water. Diluted libraries were prepared in the same fashion as the other titration methods, using a series of 5 dilutions (1:1,024,000; 1:4,096,000; 1:16,384,000; 1:65,536,000 and 1:262,144,000). One-microliter of each dilution point was used in the ddPCR-Tail mix. After droplet generation, the prepared plate was transferred to a thermocycler and submitted to the previously described PCR profiles. Results obtained were the number of molecules per microliter of input reaction. The assay was run in triplicate for a total of 15 values per library. Data were dilution-corrected and converted into molarity.

### NGS sequencing analysis

After titration, 12 pM of DNA was loaded into the flow-cell. PE-Sequencing was done in a HiSeq2500 with a read length of 100nt. The resulting sequencing data was then treated, sorted and quality controlled using FastQC Programs (Babraham Bioinformatics; Andrews S 2010. FastQC High Throughput Sequence QC Report)^27^. We report here the quality score, the total number of reads, and the individual number of reads per index for each of the titration methods. The full NGS data from the sequencing is available through NCBI SRA database under the accessing number NCBI SRA PRJNA260389.

### Availability of supporting data

The integrality of the HiSeq flowcell data from the Paired-end sequencing is available through NCBI SRA database under the accessing number NCBI SRA PRJNA260389. Data represents the total of the 8 lanes used on the flowcell (~270Go).

## Additional Information

**How to cite this article**: Robin, J. D. *et al.* Comparison of DNA Quantification Methods for Next Generation Sequencing. *Sci. Rep.* **6**, 24067; doi: 10.1038/srep24067 (2016).

## Supplementary Material

Supplementary Information

## Figures and Tables

**Table 1:** Titration methods used for Next Generation Sequencing.

Library Titration Method	Requires Bio-analyzer analysis	Quantification Method	Sensitivity	DNA Input per Lane
QuBit	yes	Estimation through Average Library Molecular Size	pg-ng	8 pM
PicoGreen	yes	Estimation through Average Library Molecular Size	pg-ng	8 pM
qPCR	yes	Estimation through Average Library Molecular Size	0.1-1 pg	8 pM
ddPCR	no	Count of number of molecules per microliter	fM	8 pM

NGS consists of massive parallel sequencing of libraries from shRNA screen to ChIPseq, Exome -, Whole-genome sequencing and chromatin capture such as HiC and 4C. This table reports the most common methods for titration, including dsDNA quantification by spectrofluoremetry (QuBit, PicoGreen); real-time PCRs (qPCR, KapaBiosystems); and finally ddPCR (BioRad). Using the advantages of an emulsion PCR approach, ddPCR-based titration, unlike the others does not require a high sensitivity electrophoresis analysis (BioAnalyzer, Agilent) in order to determine the average molecular size of the library for calculation of molarity. After accurate quantification, 8 pM–12 pM of DNA is loaded per lane in the deep sequencer (Illumina’s Recommendations).
