# Supplementary material for: Comparison of DNA Quantification Methods for Next Generation Sequencing
Source: Sci Rep. 2016 Apr 6;6:24067. doi: 10.1038/srep24067 (PMC4822169; doi:10.1038/srep24067)

Comparison of DNA Quantification Methods for Next Generation Sequencing

Jérôme D. Robin, Andrew T. Ludlow, Ryan LaRanger, Jerry W. Shay, and Woodring E. Wright

Supplemental Figures

Figure SI Comparison from TaqMan and UPL internal probe in gene expression assay.

Figure SII Comparison from UPL internal and UPL tail probe in gene expression assay.

Table SI Primer list associated with the method and probe used in the assay respectively.

Figure SIII Representative reports from the FastQC analysis.

Figure SI

Comparison from TaqMan and UPL internal probe in gene expression assay. Linear curve were determined from dilution series (7 point dilution) of gene expressions assay (hTERT), a no-template control was loaded on the 8th lane (H03 and H02, respectively) Top: Schematic representation and ddPCR readout of the two strategies and localization of fluorescent probes. Bottom Left: Linear regression associated with each method. Linear regression equations are respectively, for Taqman: R^2 =0,9999; Y = 371.4*X + 174.7 , and for UPL#52 R^2= 0,99968; Y = 373.5*X + 91.64. Bottom Right : Correlation plot of the two strategies and associated equation, goodness of fit R^2 = 0,9999, p<0.0001. No significant differences were found between the results issued from the two different strategies.

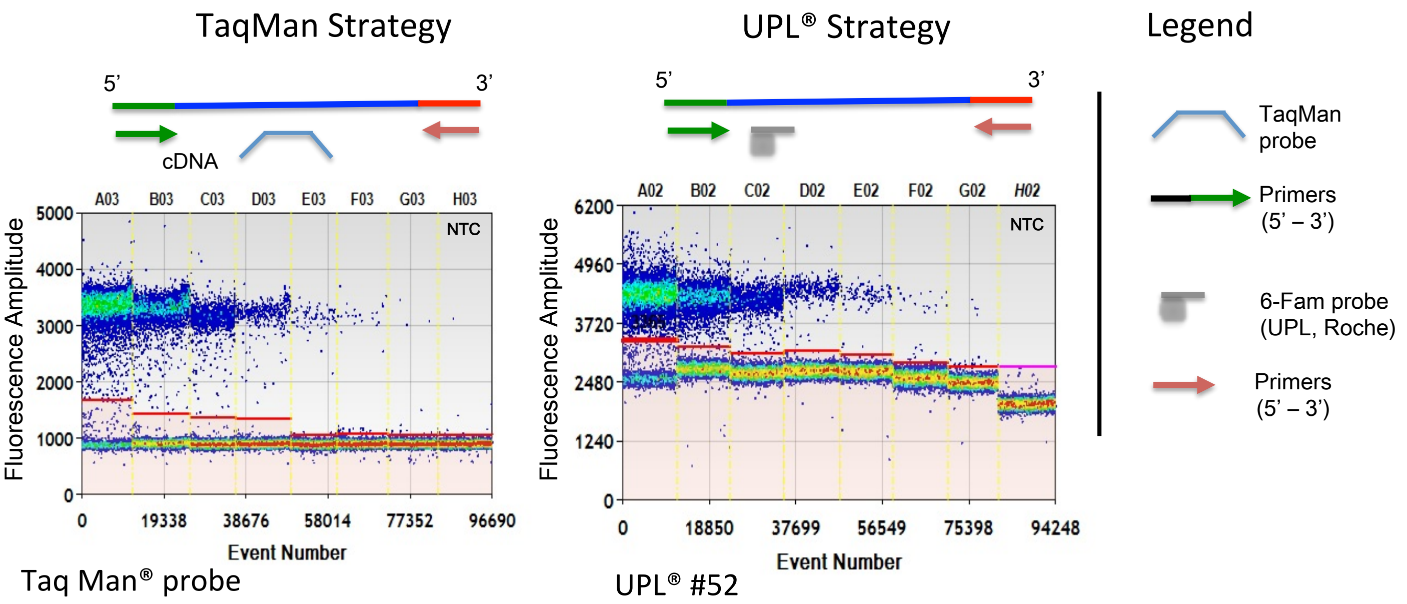

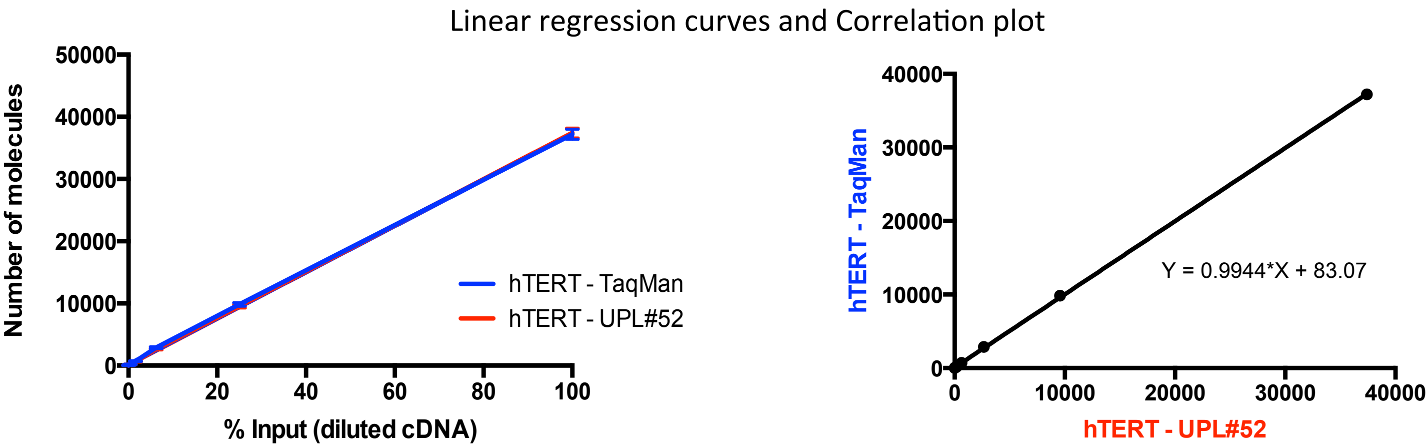

Figure SII

Comparison from UPL internal and UPL tail probe in gene expression assay. Linear curve were determine from dilution series (7 points) of gene expressions set (HPRT), a no-template control was loaded on the 8th lane (H01 and H08). Left: Schematic representation and ddPCR readout of the two strategies and localization of fluorescent probes. Right, Top: Linear regression associated with each method. Correlation values for the respective linear regression are for the internal UPL#22 $R^2 = 0.9991$; Y = 501.2*X + 368.9 and $R^2 = 0.9958$; Y = 476.1*X – 214.3 for the tail probe#52 strategy. Right, Bottom: Correlation plot of the method (Internal vs Tail) and associated equation. Goodness of fit $R^2 = 0.9923$, $p < 0.0001$. No significant differences were found between the results issued from the two different strategies.

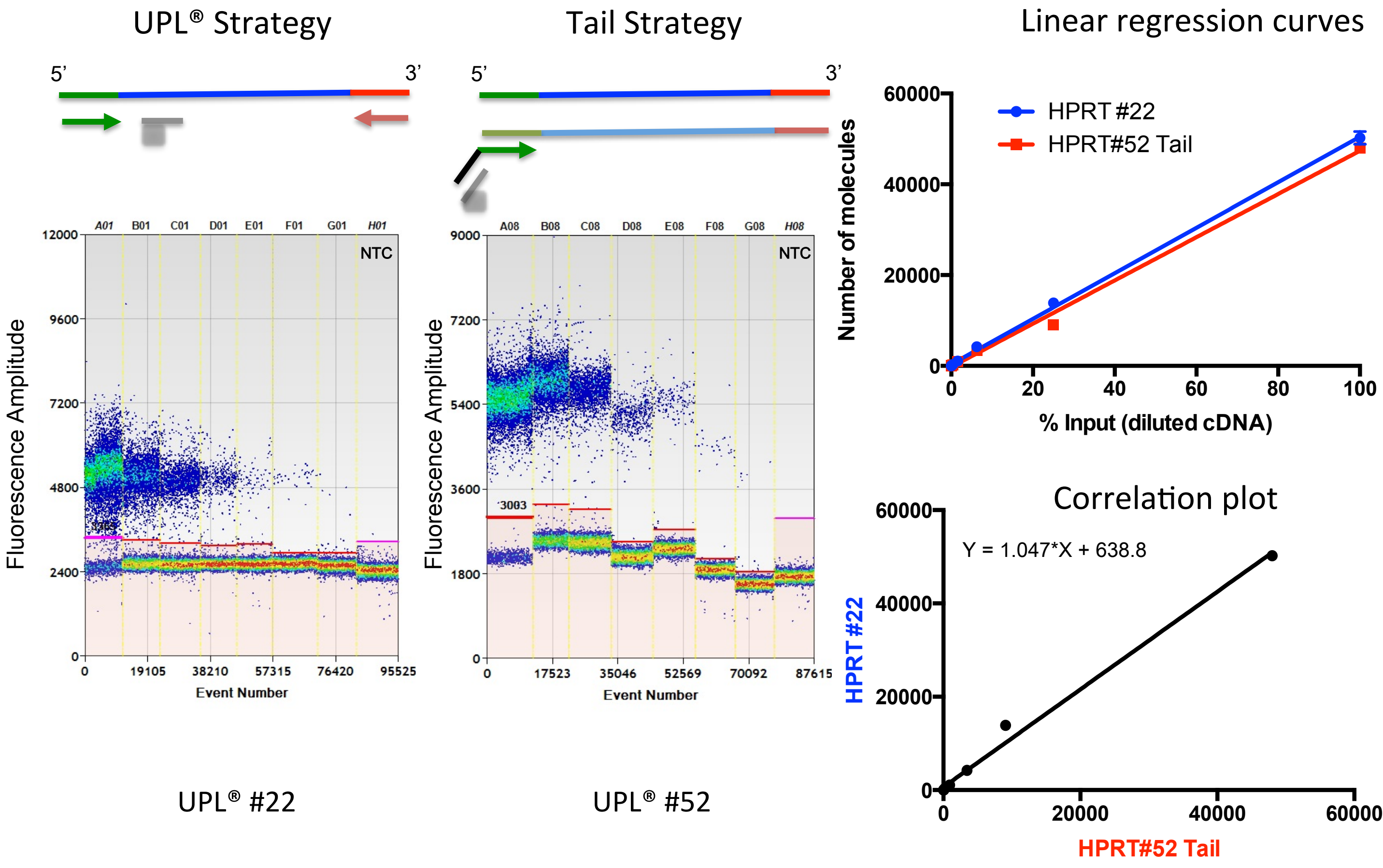

Table SI

Primer list associated with the method and probe used in the assay respectively.

	Forward Primer 5'-3'	Reverse Primer 5'-3'	Detection sequence	Detection strategy and probe type
hTERT gene expression	acagttcgtggctcacctg	gcgtaggaagacgtcgaaga	5'-6-FAM-ctcctccc-AQ-3'	UPL - #52
hTERT gene expression	acagttcgtggctcacctg	gcgtaggaagacgtcgaaga	5'HEX-acatgcgacagttcgtggctca-BHQ-3'	5' hydrolysis probe
HPRT	tgatagatccattcctatgactgtaga	caagacattctttccagttaaagttg	5'-6-FAM-tggtggag-AQ-3'	UPL-#22
HPRT-Tail	gggaggagtgatagatccattcctatgactgtaga	caagacattctttccagttaaagttg	5'-6-FAM-ctcctccc-AQ-3'	UPL - #52
NGS-Tail	gggaggagaatgatacggcgaccaccgagatctacactctttccctacacgacgctcttccgatct	caagcagaagacggcatacgagatcggtctcggcattcctgctgaaccgctcttccgatct	5'-6-FAM-ctcctccc-AQ-3'	UPL - #52

Figure SIII Representative reports from the FastQC analysis.

For each titration method used, qPCR (Sybr based, KapaBio), QuBit (fluorometer, Invitrogen), ddPCR (Taqman probe based, BioRad) and ddPCR-Tail (probe based), respectively, we provide examples of the FastQC report (Babraham Bioinformatics) attached to the sequencing. A sequential set of statistics quality tools are used for each method in order to decipher the overall quality of the dataset. In short, for all sequencing methods we report the

- 1 Per base sequence quality – represents a global view of the quality values associated for each base called across each position of the sequence (quality of the individual 100bp read). The Y axis refers to the quality score, the X the position of the base within the read. The Y axis is zone-color-coded, with green (very good), orange (average) and red (poor) as a quick mean to show the quality of the base call. For each base analysis, the blue line shows the mean, the red bar the median, the yellow boxes the inter-quartile range and whiskers the 90% interval confidence.
- 2 Per sequence quality score – shows the average quality of the full sequences, it allows one to determine if a subset of sequences are presents in the dataset.
- 3 Per base sequence content – represents the portion of each nucleotide (A,T,G,C) at each position of the sequence. The ratios of A:T and G:C are 1 and 1 respectively in normal DNA. Strong deviations from this ideal state can arise from multiple reasons such as : overrepresented sequences, indexes or technical bias, making this a strong internal control.
- 4 Per base GC content – shows the GC amount at each position of the sequence, and thus the overall GC content of the genome sequenced. This value should stay stable throughout the sequencing.
- 5 Per sequence GC content – reports the GC content across the full sequence, and compares it to an hypothetical normal distribution using the previous value as modal/ central. Distributions should overlap.
- 6 Per base N content – represents for each position in the sequence the inability of the reader to call for a specific nucleotide and its replacement by an N. This is usually seen at the ends of sequencing reads, but can also detect error-prone regions in sequencing.
- 7 Sequence length distribution – shows the distribution of sequence sizes from the dataset.
- 8 Duplicate sequences – reports the overall duplication level of a subset of sequences (200,000) from the set, and plot their relative abundance. Thus showing the degree of repetitive sequences; the number reported correspond to an estimation of the percentage of non-unique sequences.
- 9 -K-mer content – reports the presence of overrepresented small sequences within the whole dataset (at each position). In our set-up, the K-mer analysis detects the presence of the indexes use to partition the lane in 6.

Complementary information regarding the analysis modules are accessible through the Babraham Bioinformatics website. The full analysis reports of all indexes sequenced are available upon request.

<u>qPCR strategy – FastQC representative analysis report</u>

1-Per base sequence quality

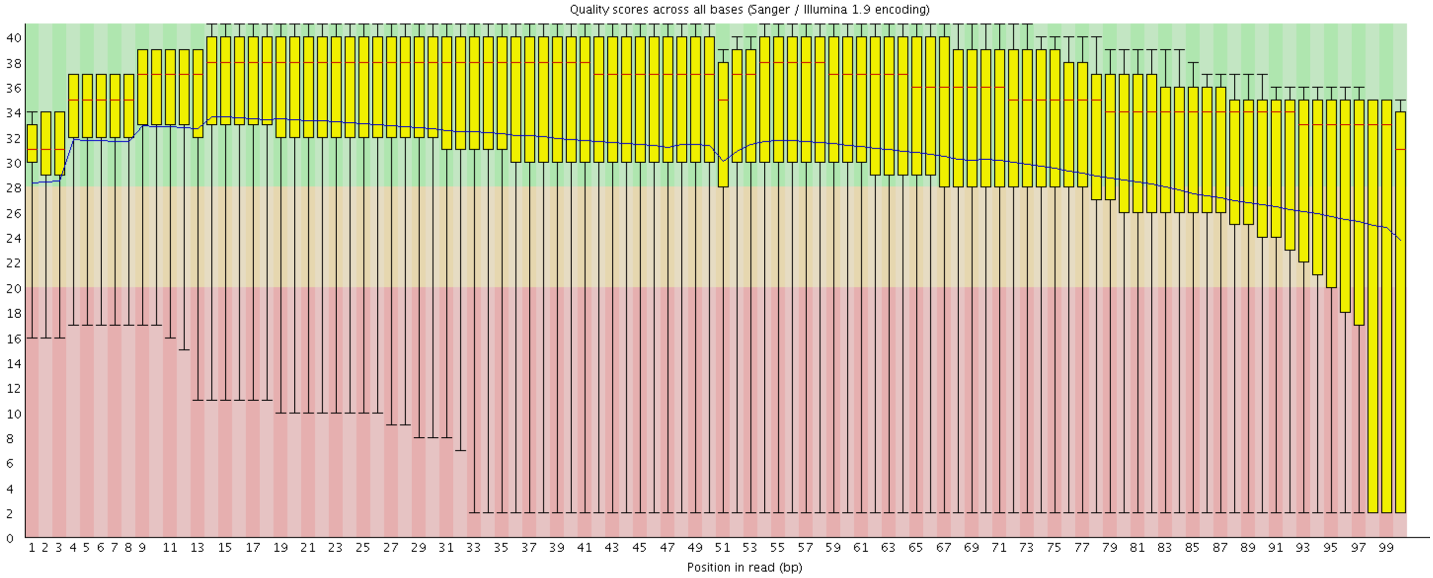

2-Per sequence quality score

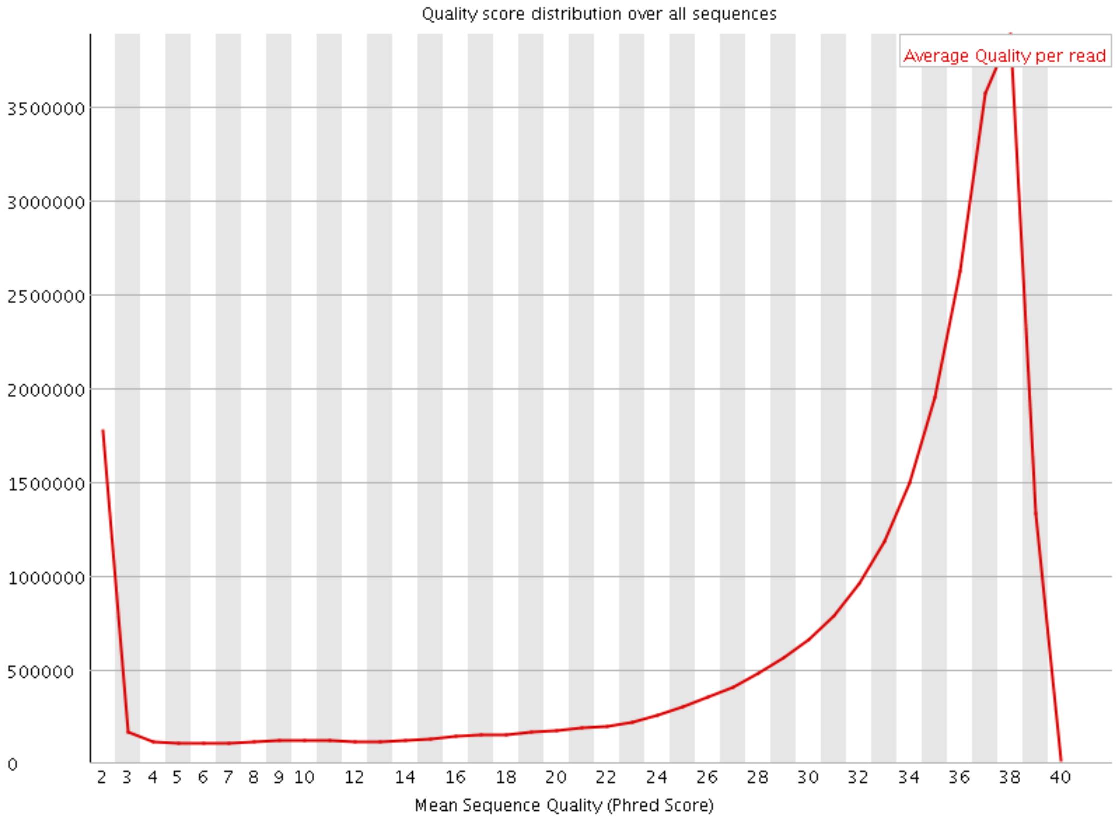

3-Per base sequence content

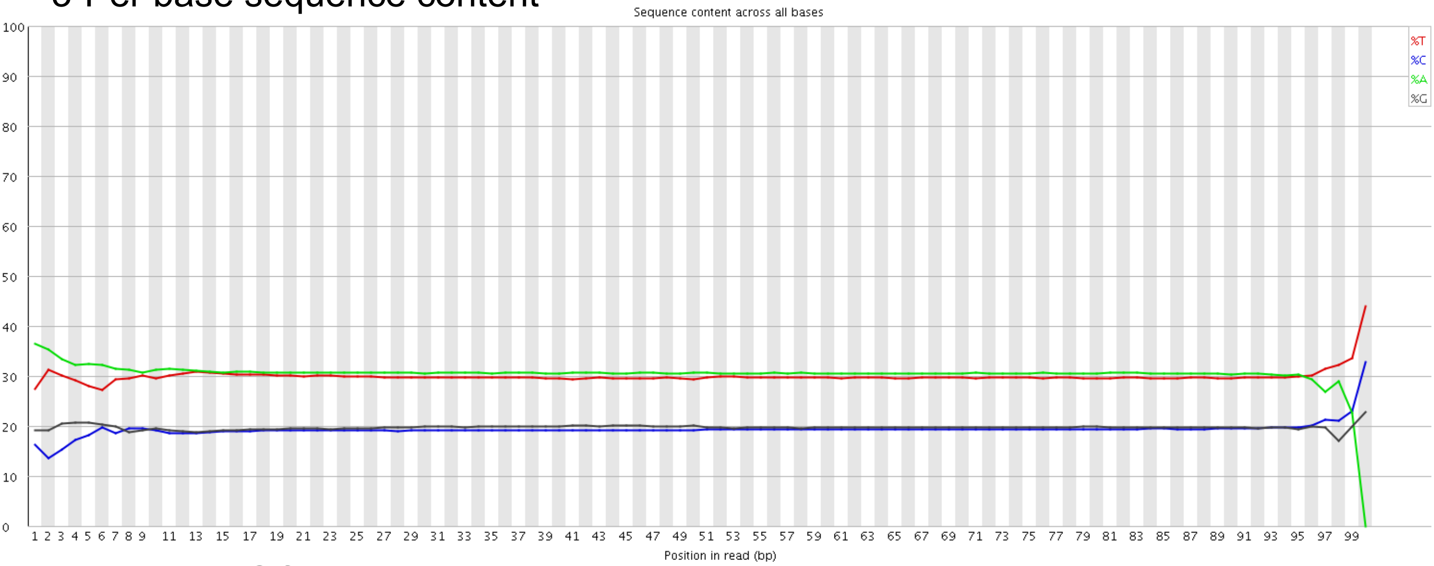

4-Per base GC content

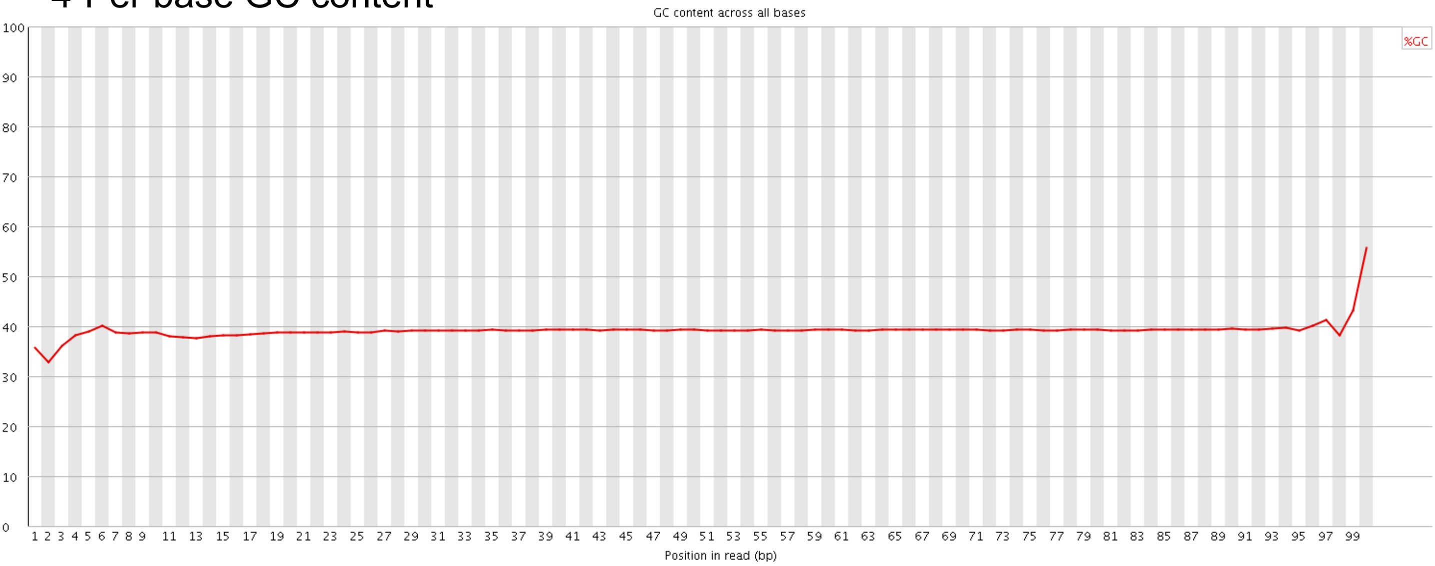

5-Per sequence GC content

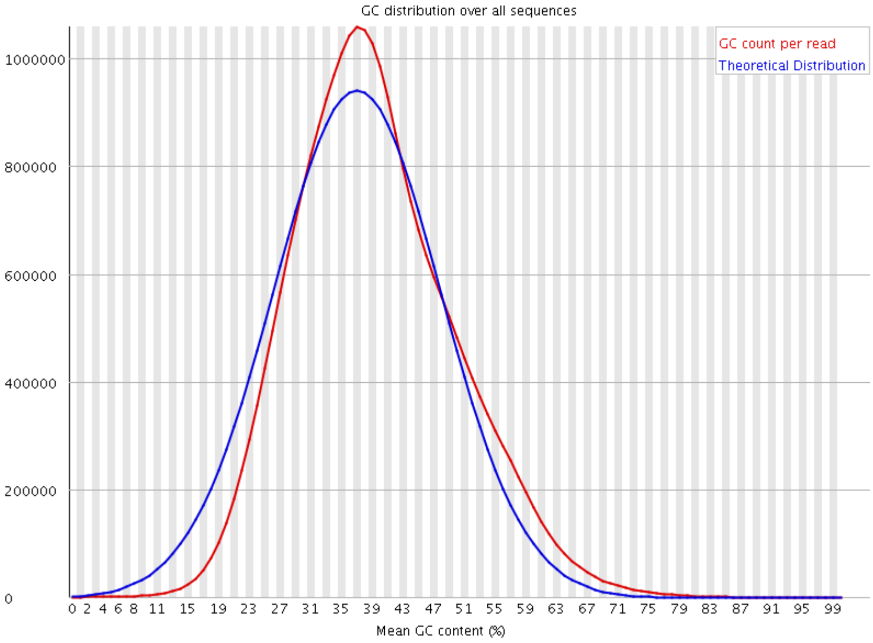

6-Per base N content

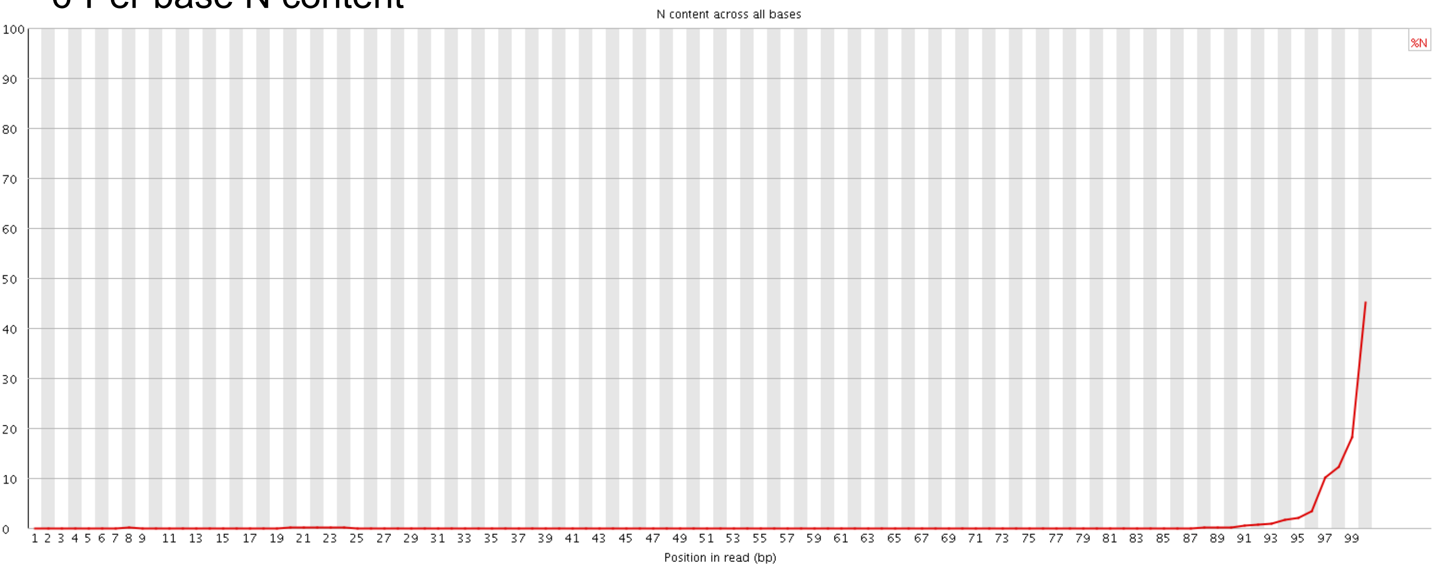

7-Sequence length distribution

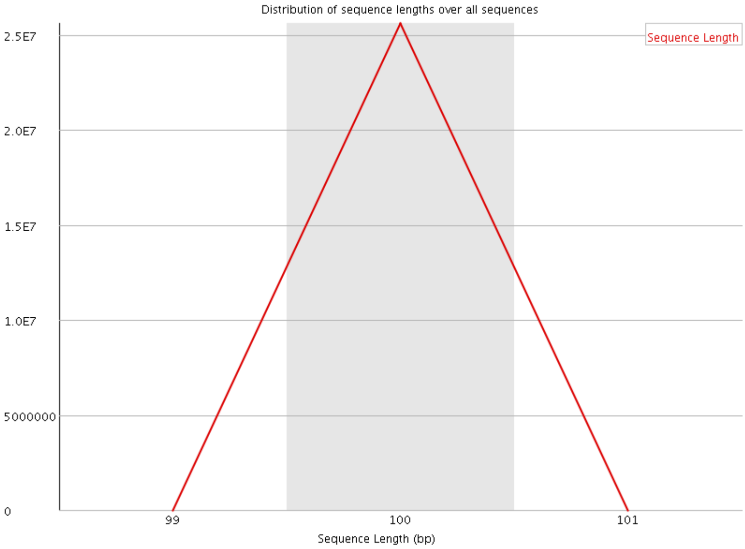

8-Sequence duplication levels

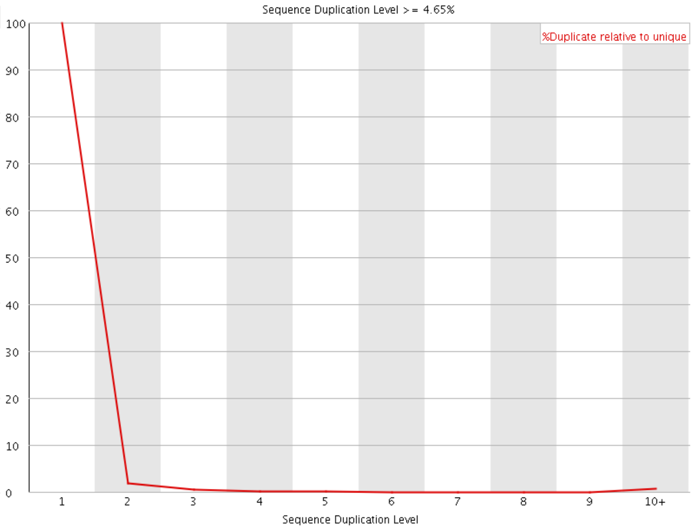

9-Kmer content

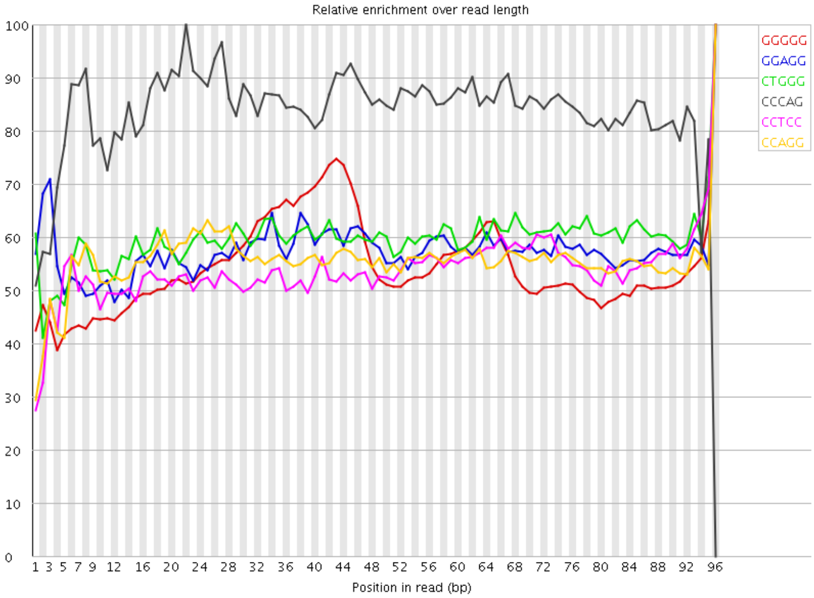

QuBit strategy – FastQC representative analysis report

1-Per base sequence quality

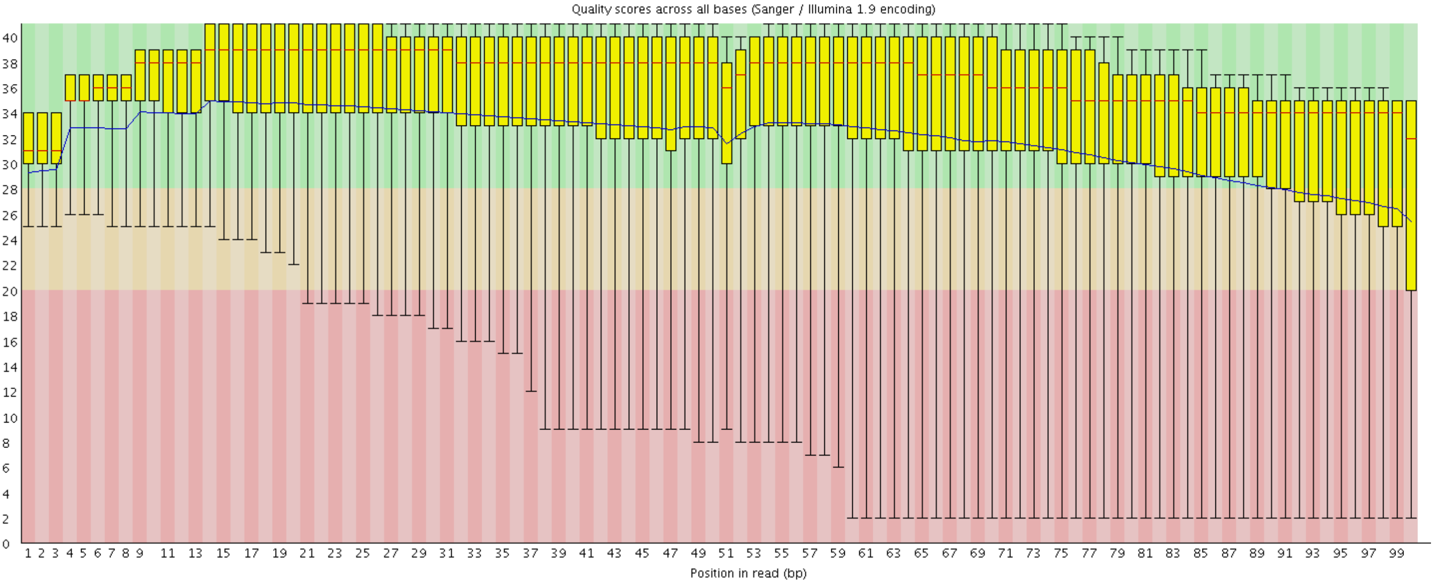

2-Per sequence quality score

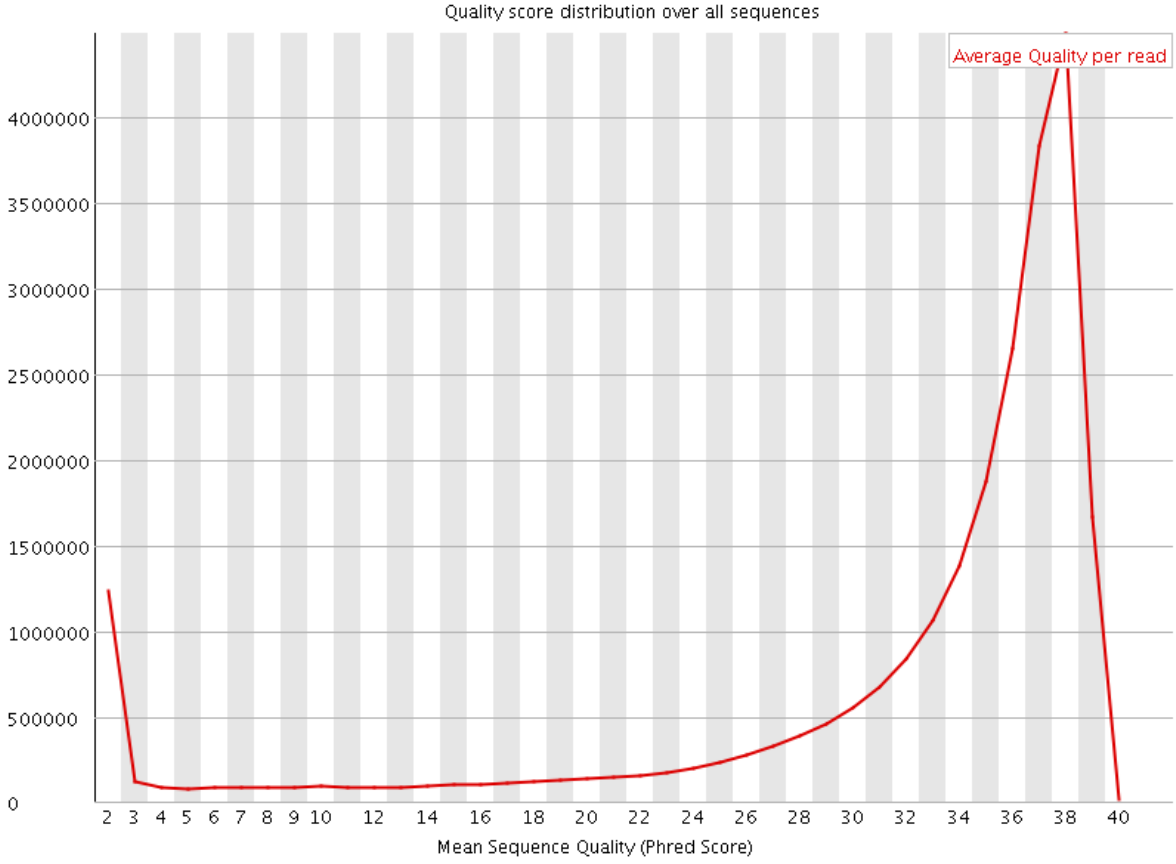

3-Per base sequence content

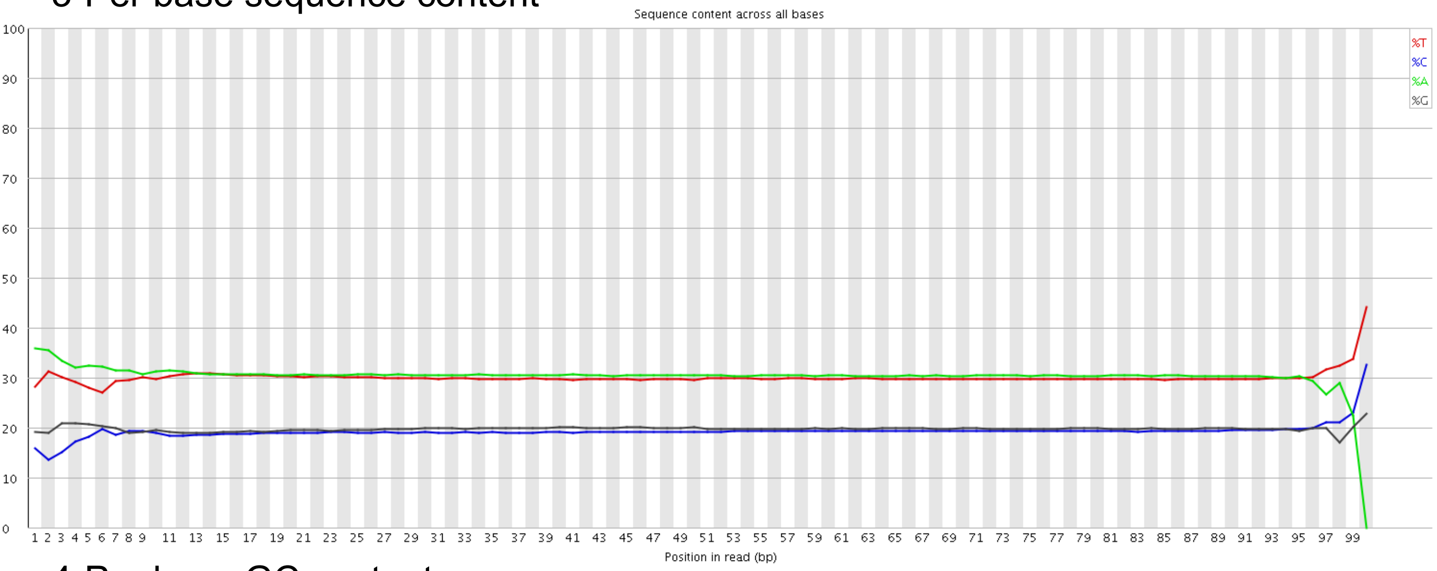

4-Per base GC content

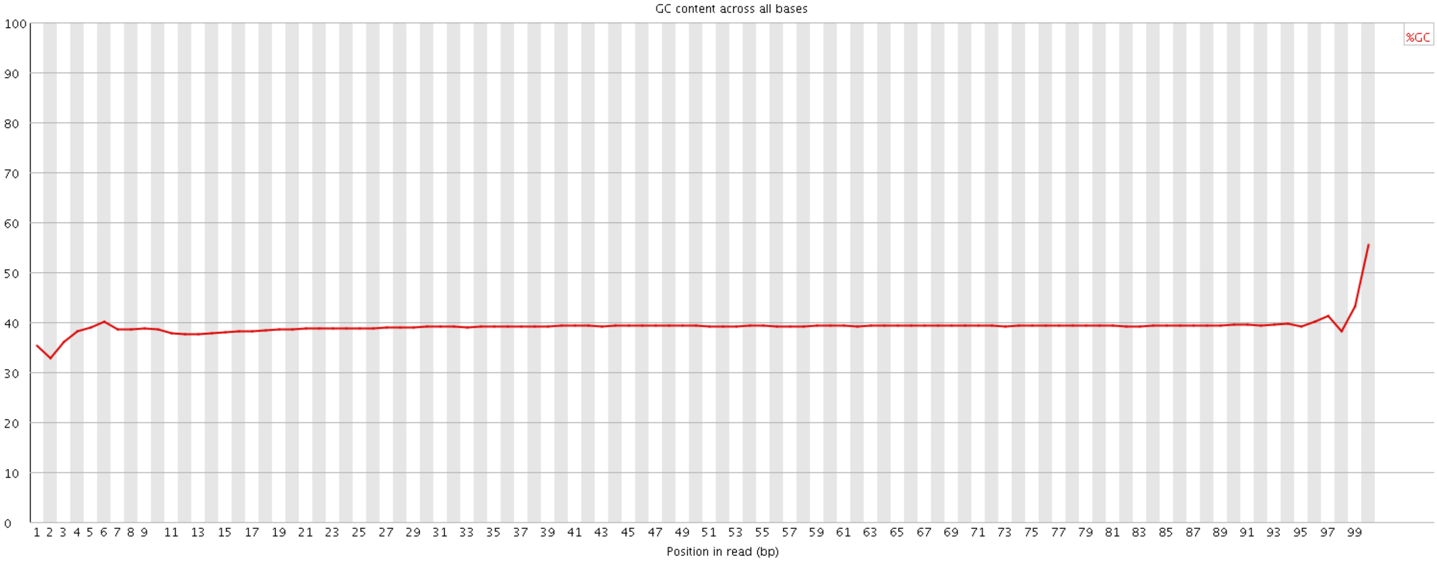

5-Per sequence GC content

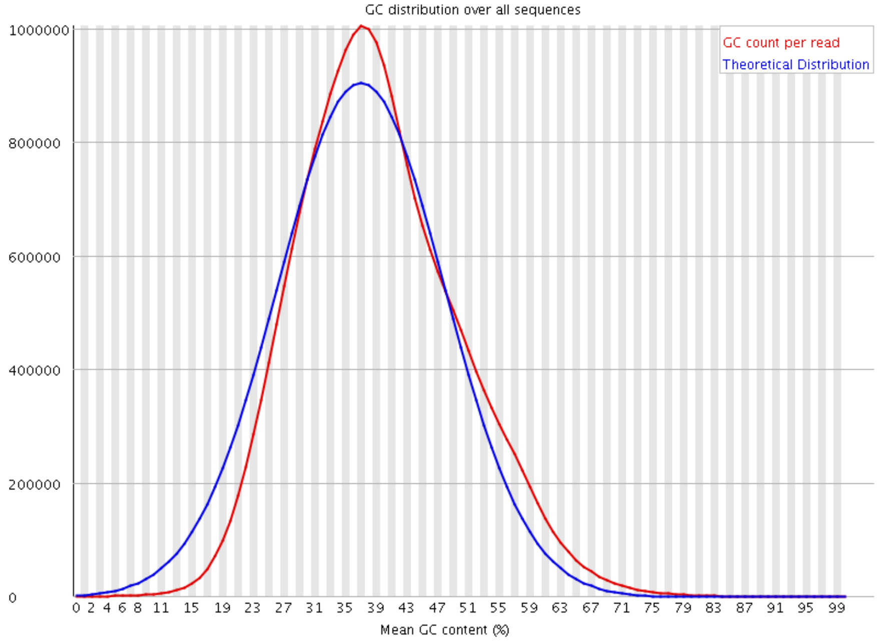

6-Per base N content

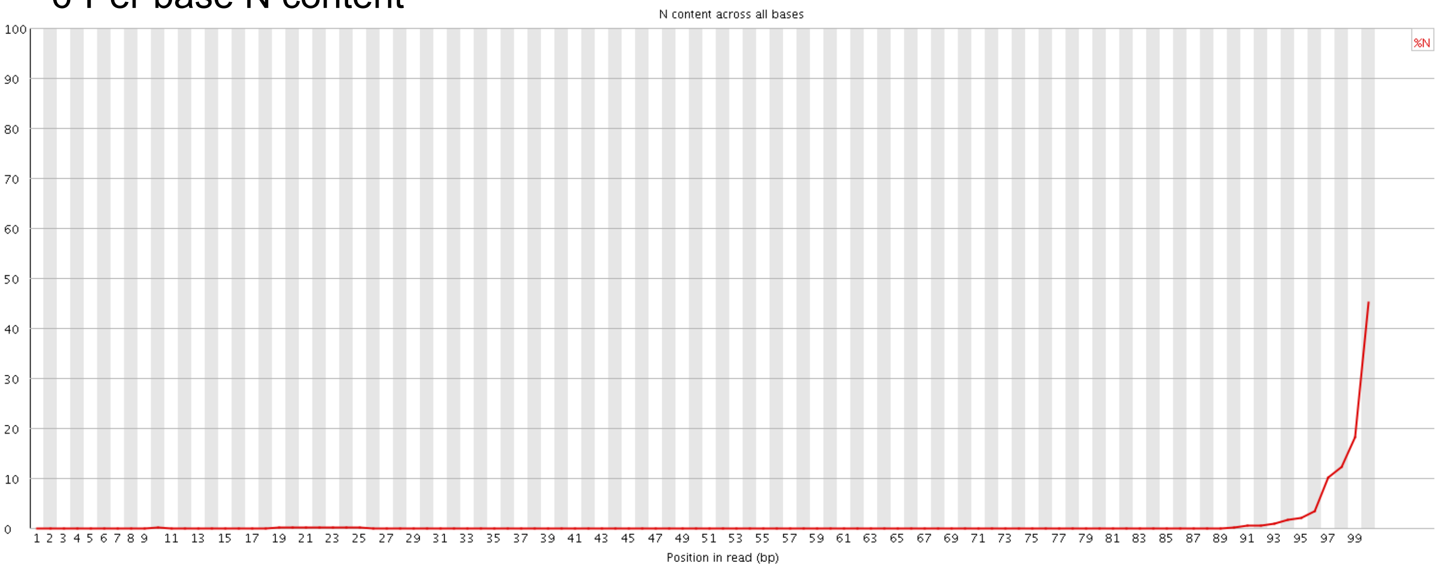

7-Sequence length distribution

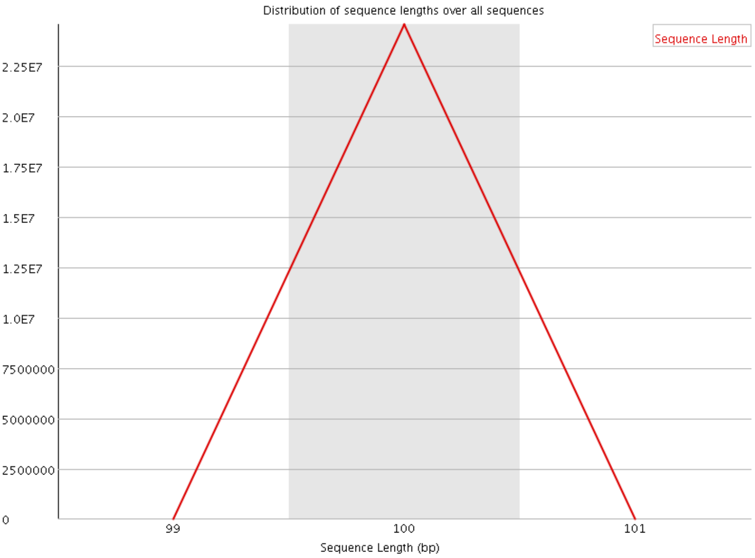

8-Sequence duplication levels

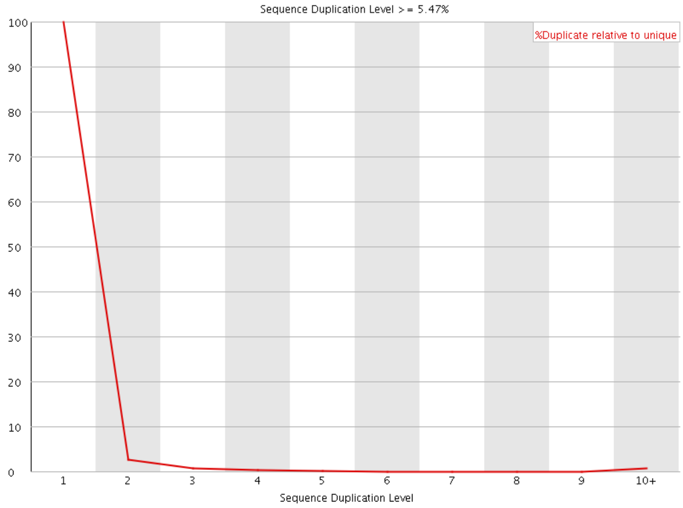

9-Kmer content

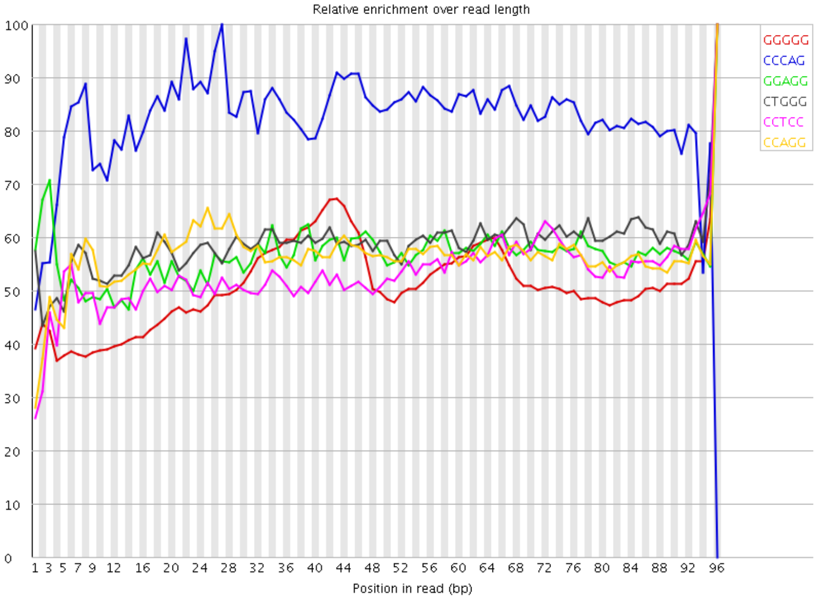

ddPCR strategy – FastQC representative analysis report

1-Per base sequence quality

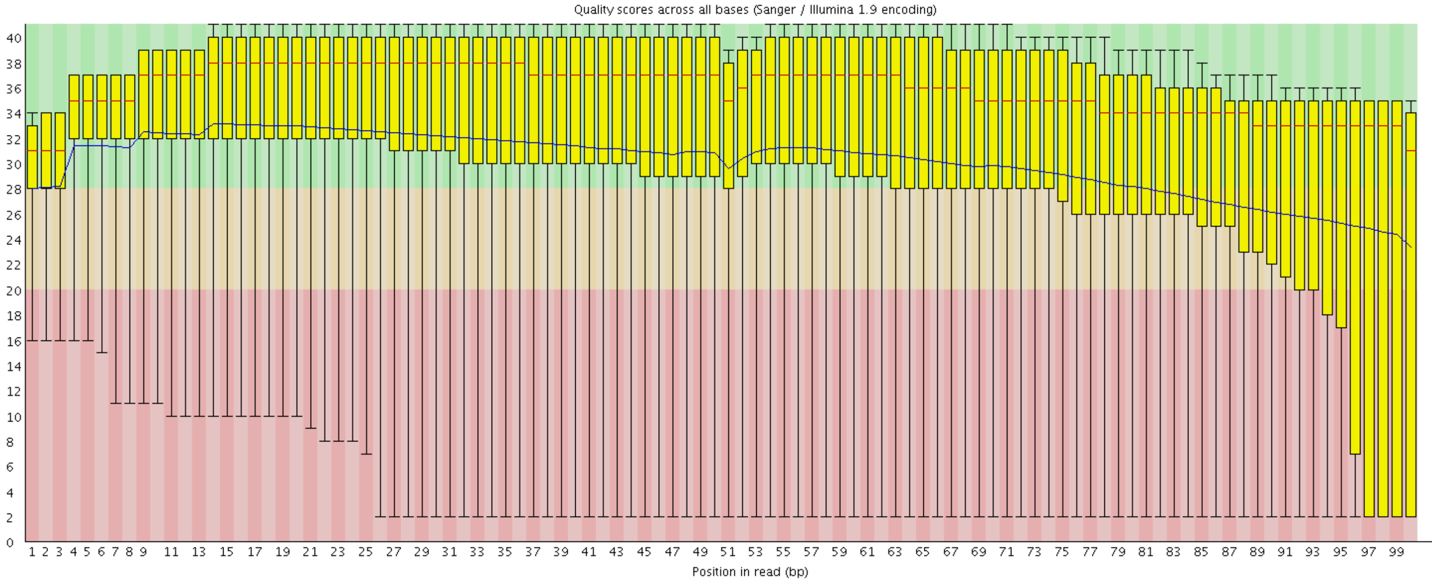

2-Per sequence quality score

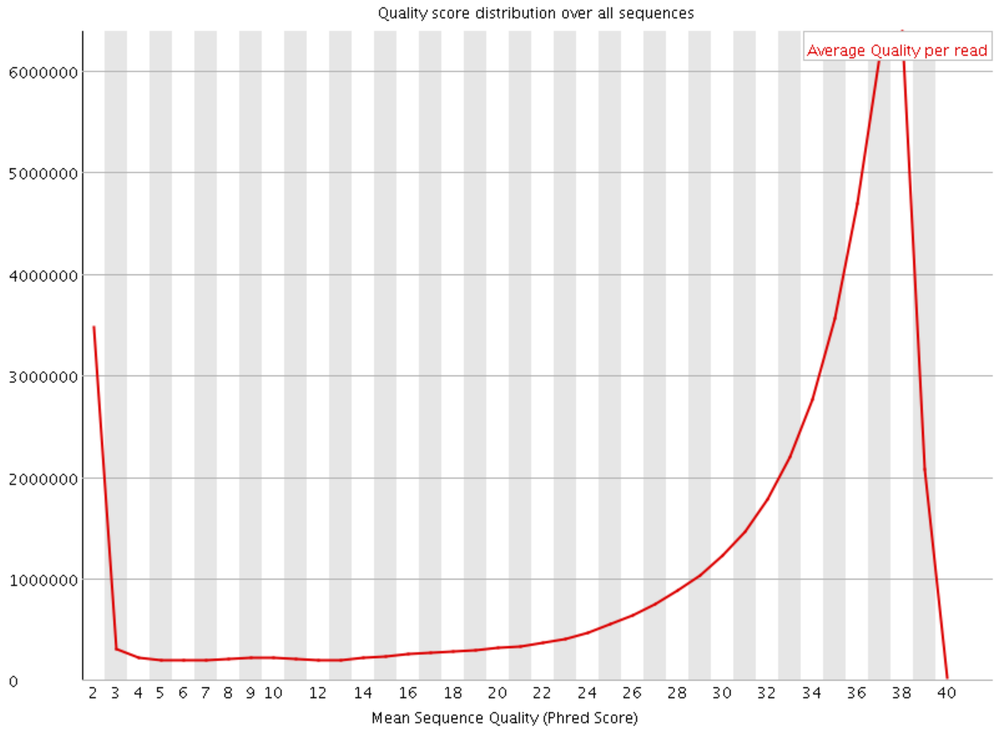

3-Per base sequence content

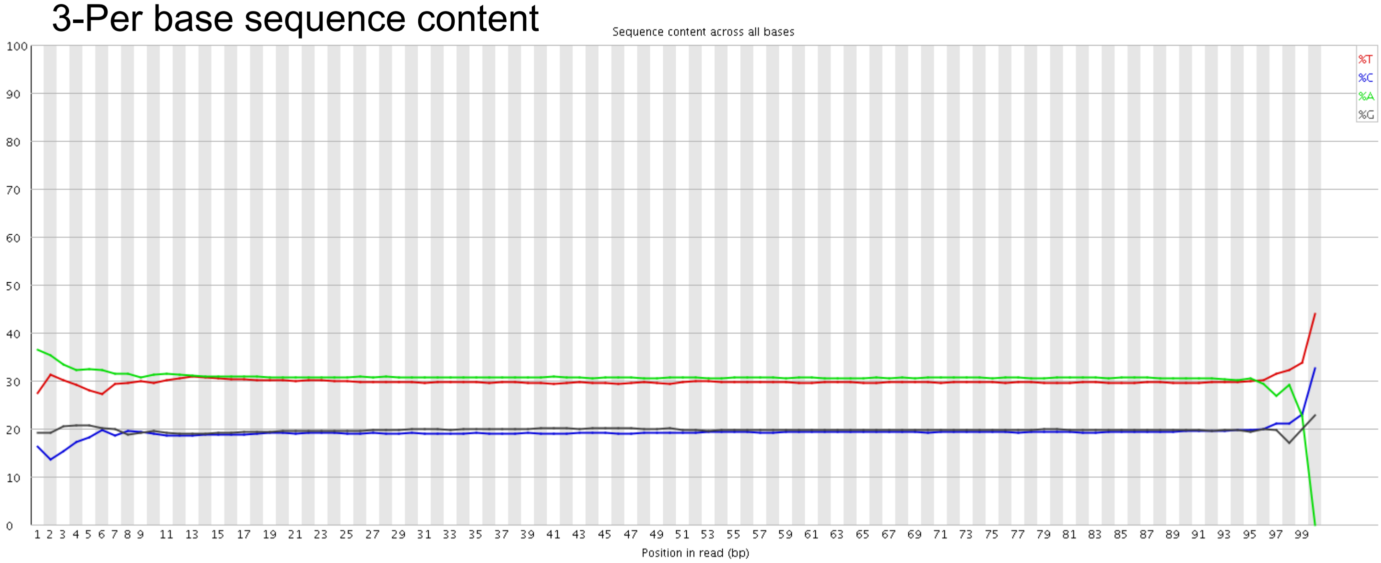

4-Per base GC content

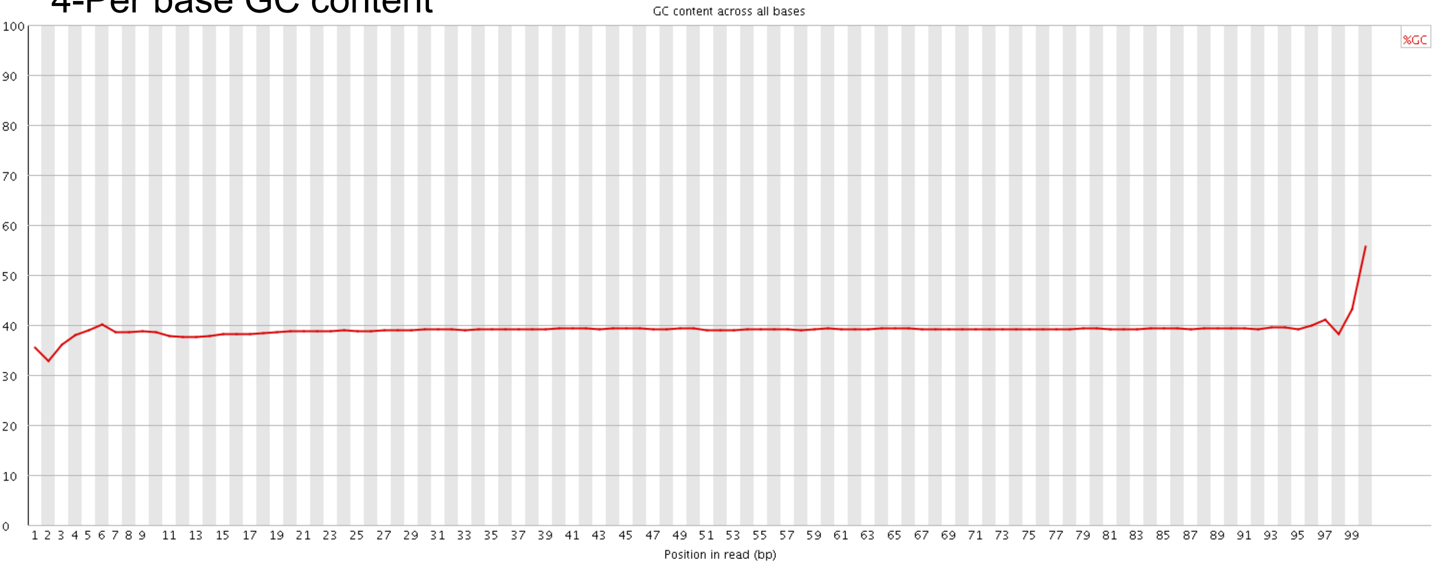

5-Per sequence GC content

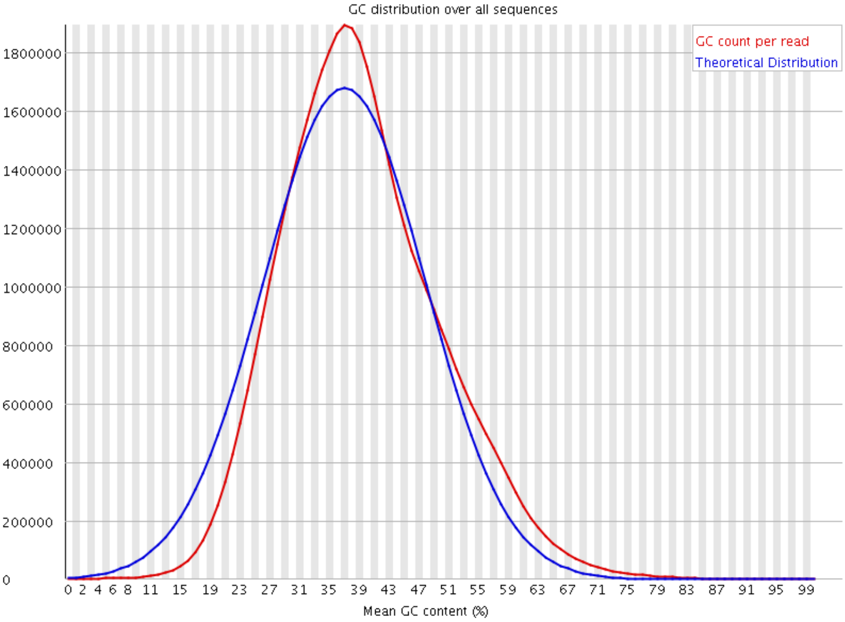

6-Per base N content

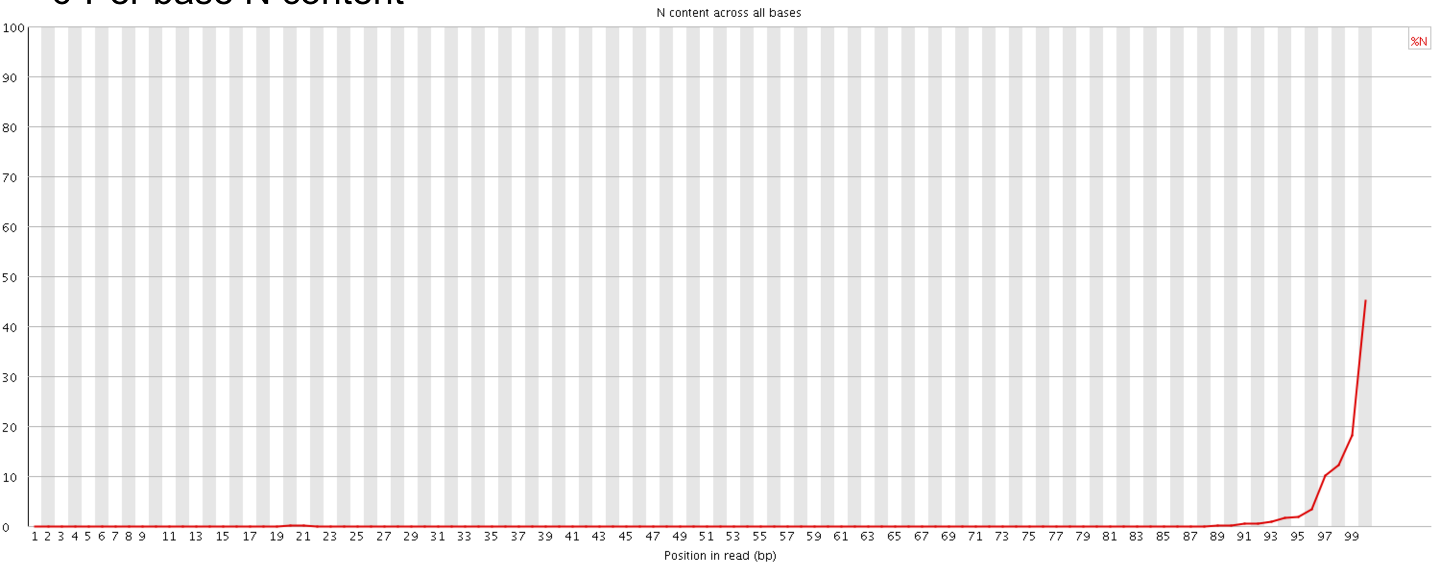

7-Sequence length distribution

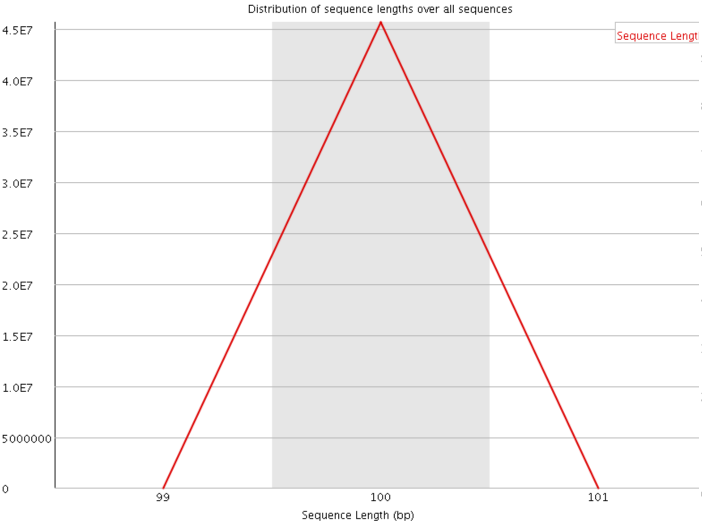

8-Sequence duplication levels

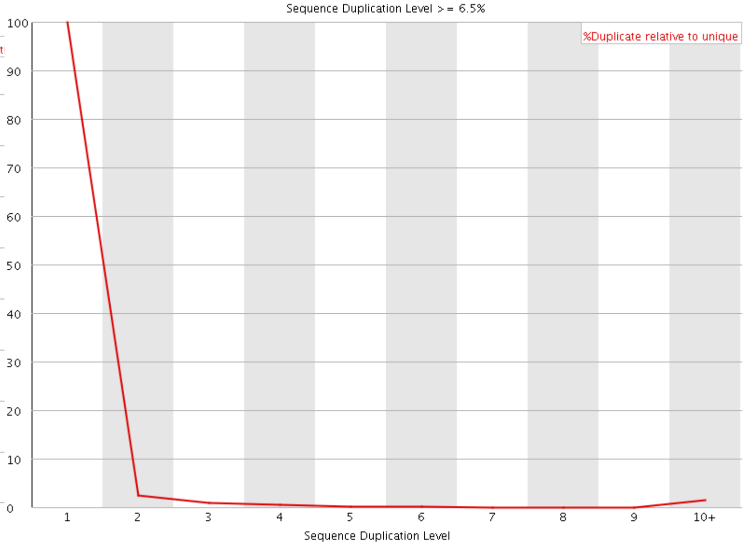

9-Kmer content

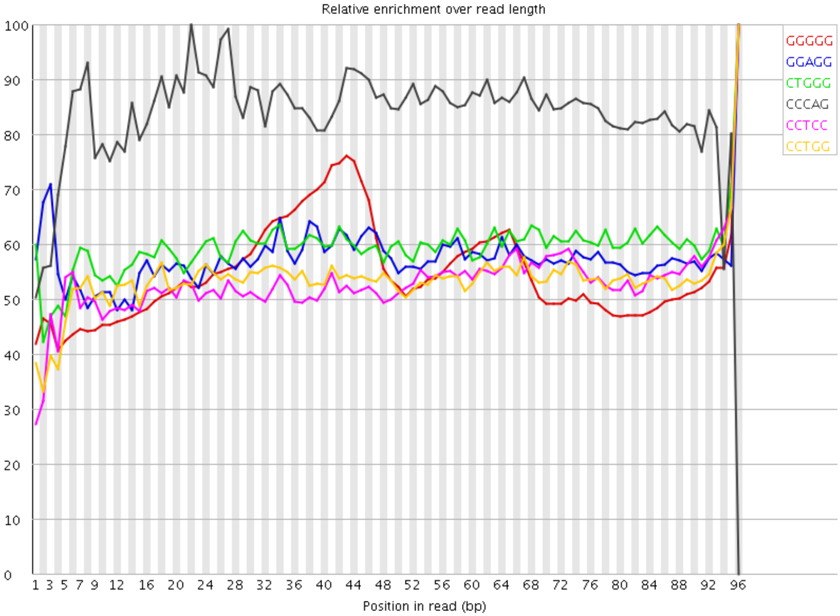

ddPCR-Tail strategy – FastQC representative analysis report

1-Per base sequence quality

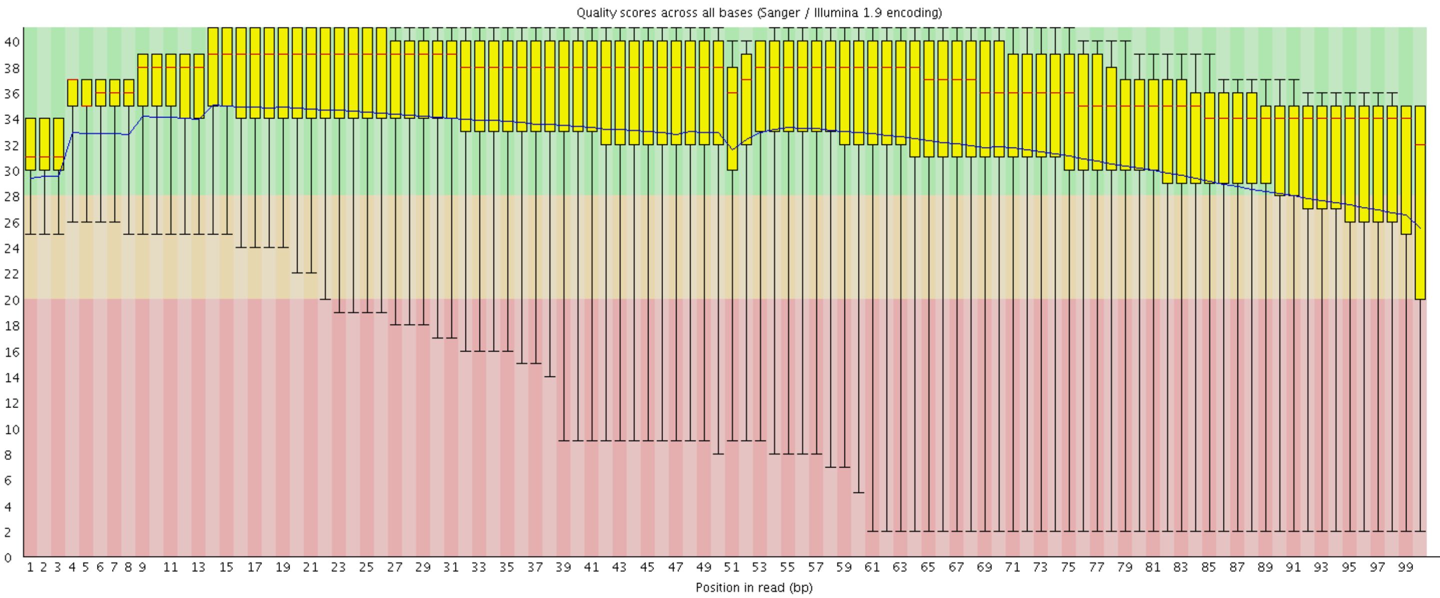

2-Per sequence quality score

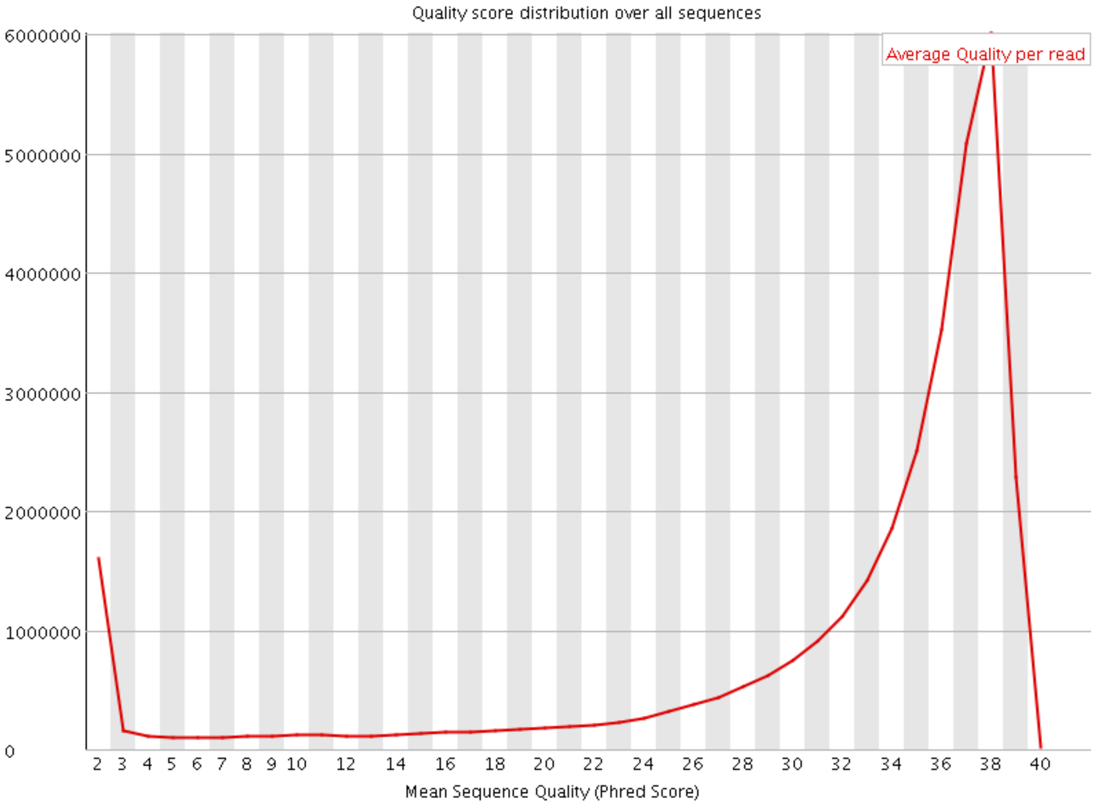

3-Per base sequence content

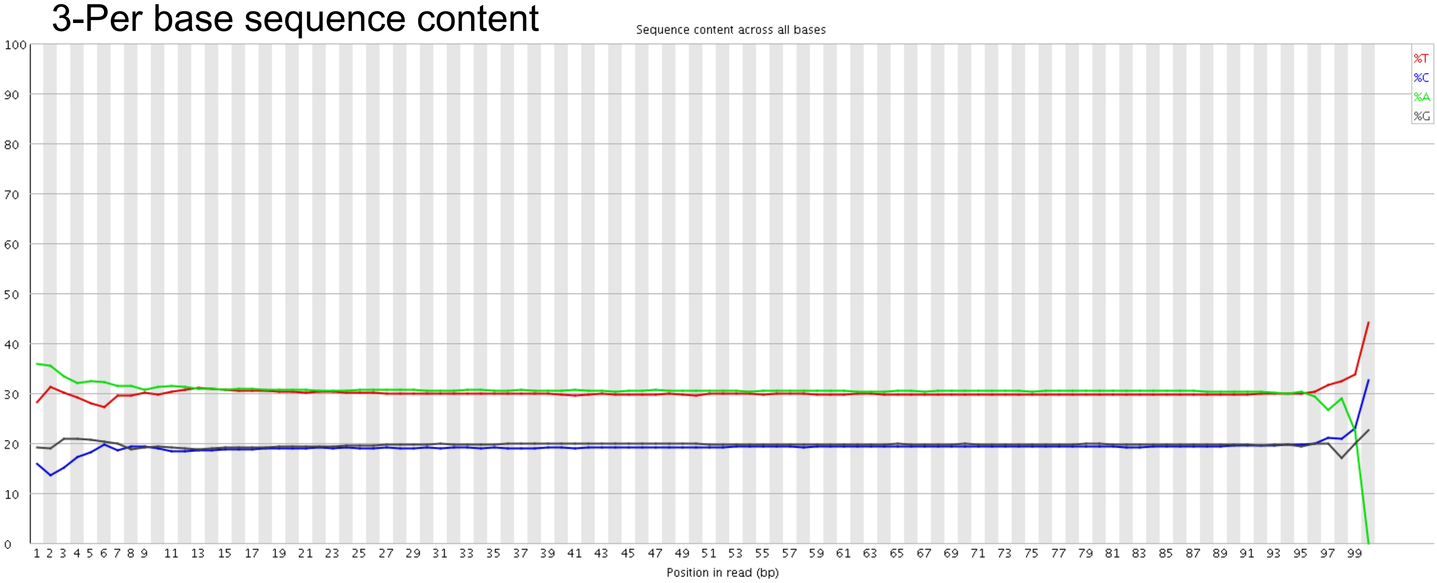

4-Per base GC content

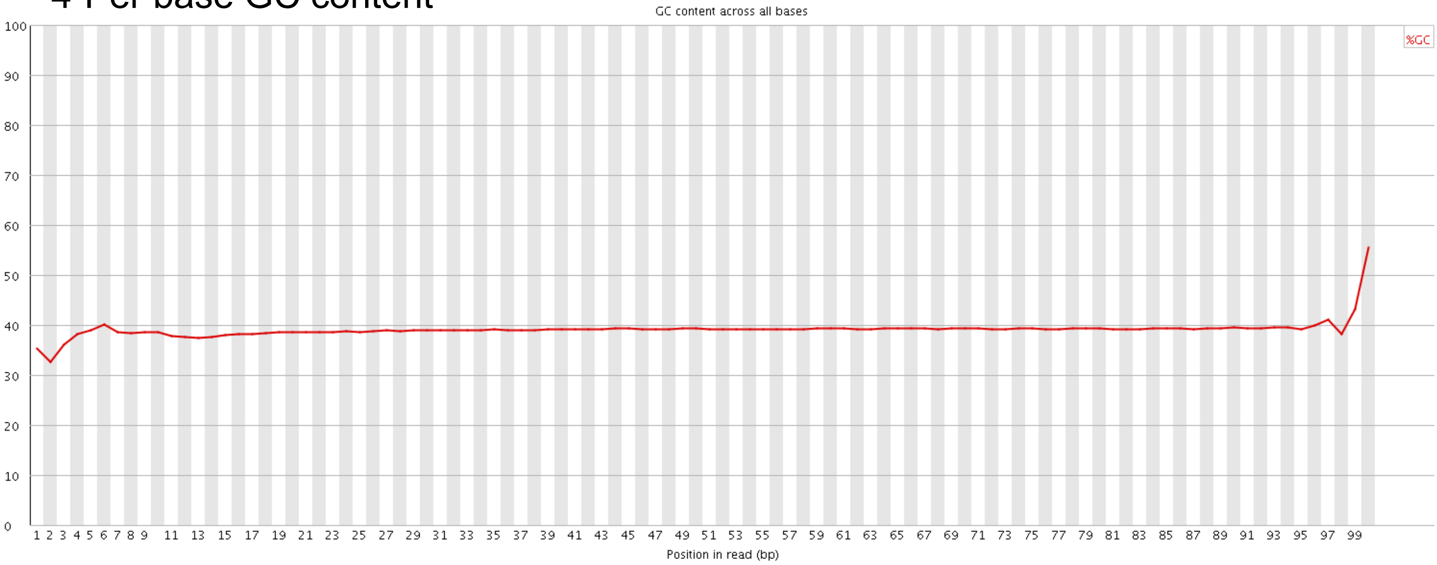

5-Per sequence GC content

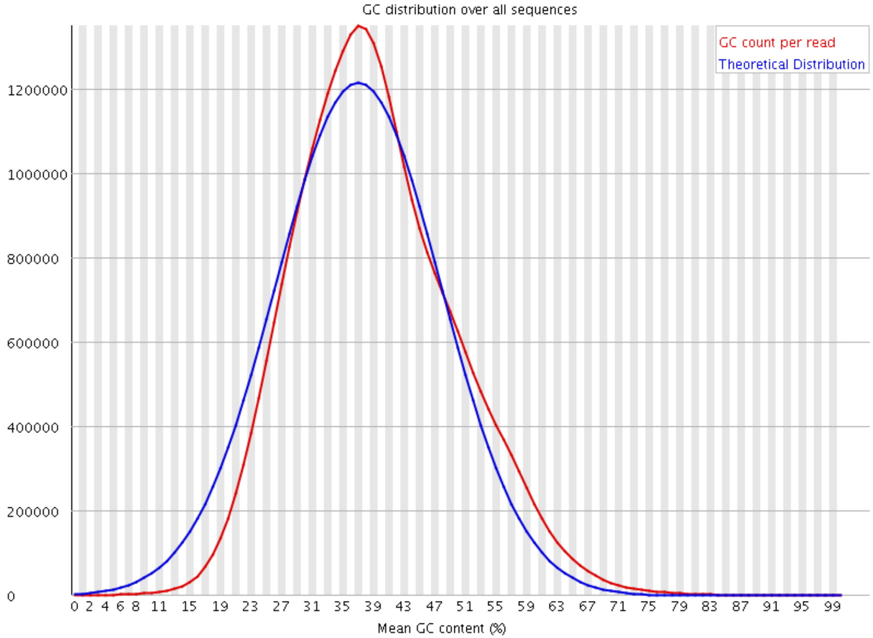

6-Per base N content

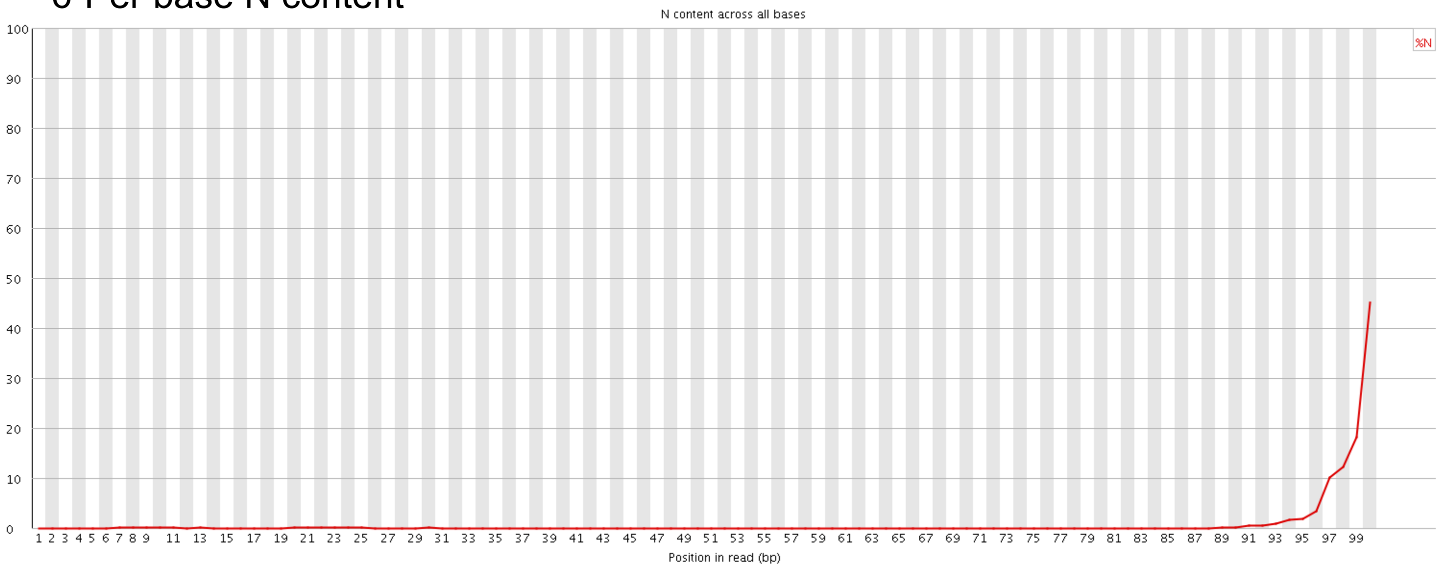

7-Sequence length distribution

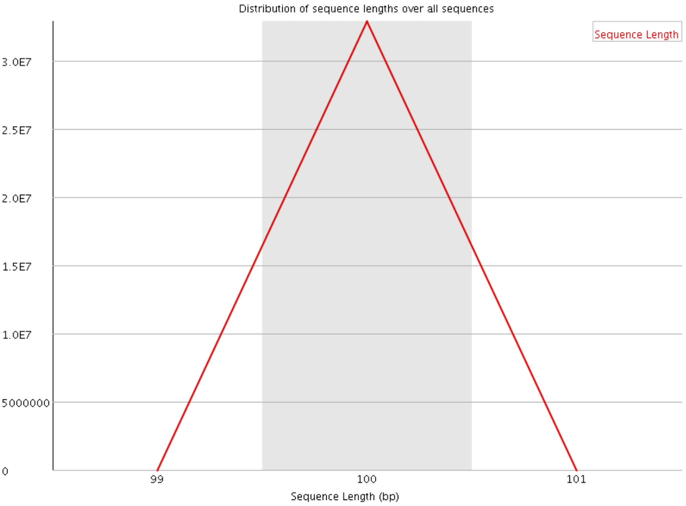

8-Sequence duplication levels

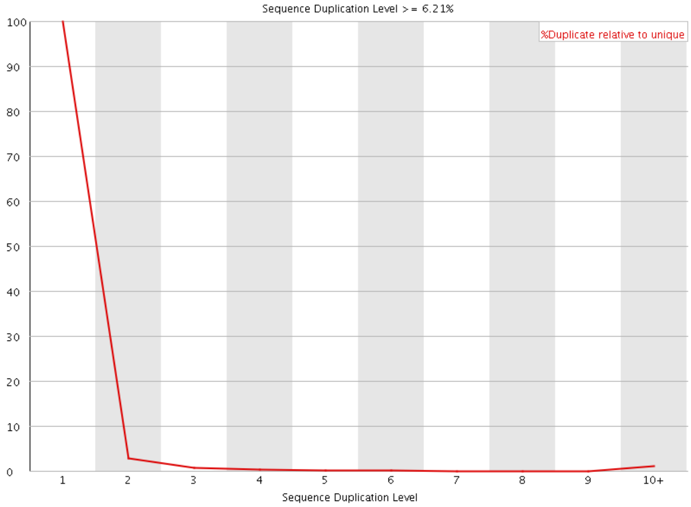

9-Kmer content

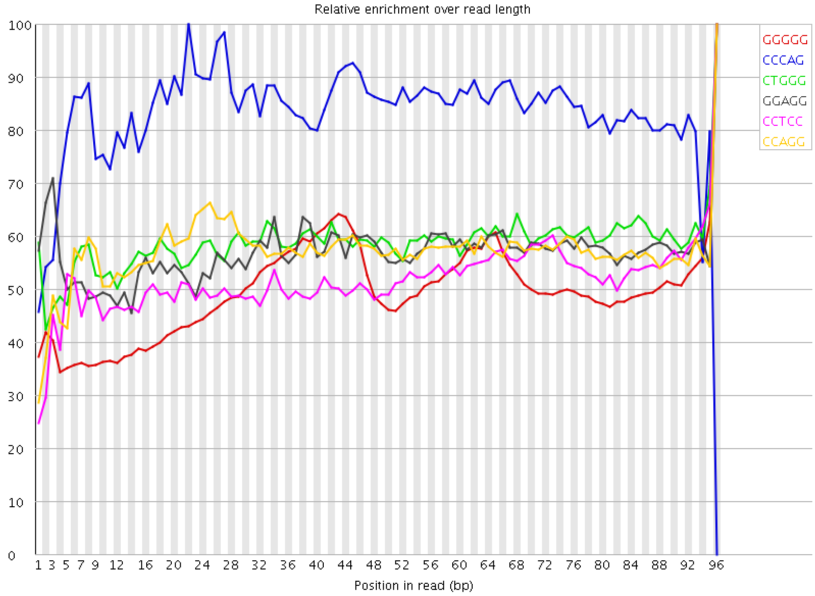

Supplement: Supplementary Information [file srep24067-s1.pdf]
